# Food and Nutrient Intakes Assessed with Dietary Records for the Validation Study of Self-administered Food Frequency Questionnaire in JPHC Study Cohort I

**DOI:** 10.2188/jea.13.1sup_23

**Published:** 2007-11-30

**Authors:** Satoshi Sasaki, Tosei Takahashi, Yoji Iitoi, Yasuhiko Iwase, Minatsu Kobayashi, Junko Ishihara, Masayuki Akabane, Shoichiro Tsugane

**Affiliations:** 1 Epidemiology and Biostatistics Division, National Cancer Center Research Institute East.; 2 Department of Nutrition, Tokyo University of Agriculture.; 3 Department of Applied Bioscience, Tokyo University of Agriculture.; 4 Cancer Information and Epidemiology Division, National Cancer Center Research Institute.

**Keywords:** weighed dietary record, nutrient, food group, diet

## Abstract

We present here the survey methods and basic results of dietary records which were used as reference values in the present validation study of a self-administered food frequency questionnaire (FFQ) for the 5-year follow-up survey of the JPHC study. A semi-weighed dietary record was kept for four seven consecutive days in each of the four seasons in 3 areas, i.e., Iwate, Akita, and Nagano, and for seven consecutive days in both winter and summer in Okinawa. The mean intakes were significantly different between areas for some nutrients and food groups. A significant seasonal difference in the mean intakes was also observed in some nutrients such as carotene and vitamin C, and in some food groups such as potatoes, vegetables, and fruits in both sexes, and alcoholic beverages in men and milks in women (p<0.001).

Food and nutrient intakes have frequently been used as the "gold standard" in several validation studies of newly developed dietary assessment questionnaires. In this validation study of a self-administered semiquantitative food frequency questionnaire (FFQ) used in the 5-year follow-up survey of the JPHC study, 28-day dietary records (14-day records in Okinawa) were collected from the sample populations living in 4 cohort areas (Iwate, Akita, Nagano, and Okinawa). In this paper, brief computation methods and the area-based intake levels of main food groups and nutrients are presented as basic data for the subsequent results of the validation study. The basic results on seasonal variation were also presented for nutrients and food groups.

## METHODS

### Data Collection

Semi-weighed dietary records of four different seasons over seven consecutive days were collected by a method used in the National Nutrition Survey (NNS)^1^ with some modifications. Research dietitians instructed the subjects to record all foods and beverages prepared and consumed in a specially designed booklet. The participants were asked to provide detailed descriptions of each food, including the methods of preparation and recipes whenever possible. The dieticians checked the records at each participant's home during the survey and reviewed them in a standardized way after recording.

### Coding

All foods ingested were coded with food codes by trained dietitians mainly in one laboratory. Some cooked foods and menus for which it was difficult to estimate the food items used in the preparation were coded with prepared cooked foods/menu codes by the authors and converted into food items after the coding using the standard recipe database developed by the authors. For rice, well-milled rice was used for the computation. Water including cooking water such as water added for rice-boiling and for miso (fermented soybean paste)-soup was not included in the coding. The weight in grams was also coded for all foods ingested except for cooking water and some beverages. Dietary supplements were coded using temporary codes prepared by the authors, but the weights were not coded.

### Computation of Food and Nutrient Intakes

Nutrient intakes were calculated using the Standard Tables of Food Composition in Japan, the 4th revised edition^2^ for energy and 16 nutrients. As the food composition table for cholesterol has numerous missing values,^3^ one was developed applying the substituting methods used for the development of fatty acid food composition table.^4^ Cooking oils were coded as "vegetable oil" without specific type and/or brand name. For the calculation of fatty acid intakes, "vegetable oil, mixed" (mixture of 30% of rapeseed oil and 70% of soybean oil) was used for all the cooking oils appearing in the data. Rice was converted into boiled rice multiplied by 2.25 for comparison with the results obtained from FFQ.

Green and yellow vegetables were defined as 44 vegetables with 600 micrograms of carotene per 100 gram and 10 frequently used vegetables that contribute to the intake of carotene among Japanese, according to the definition by the Ministry of Health, Labor and Welfare. Additionally, mugwort (leaves) with 3600 micro grams of carotene per 100 gram food portion was included in green and yellow vegetables because it was not negligible as a carotene source among the subjects in Okinawa in a previous survey^5^. Pickled vegetables were also computed separately from total vegetables. Pickled plum was included in the pickled vegetables, whereas it was otherwise categorized as a fruit.

Among non-alcoholic beverages, only green tea brewed at home was reported without the number of cups drunken. The amount consumed was taken to be 200 g each time.

### Statistical Analysis

The 215 subjects (102 men and 113 women), both with FFQ for the validation study and the complete DR (14-day records in Okinawa and 28-day records in the other 3 areas), were included in this analysis. Mean intakes by PHC area were presented, and the difference among the 4 areas was examined using analysis of variance (ANOVA). In order to examine a possible seasonal variation of food and nutrient intakes, the mean intakes by season are also presented, and the seasonal difference was examined using two-way ANOVA adjusting for area difference. In this analysis, the data from Okinawa were excluded because of the lack of data for the survey in spring and autumn. The comparison of mean intakes between winter and summer was performed including all 4 areas using paired t-test. Intakes of nutrients and foods were adjusted for energy intake using the energy density method to eliminate possible association between intakes and total caloric intakes. The top 20 foods for each nutrient were listed, and their contributions to the nutrient were calculated.

### Data for Comparison

In order to examine different intake levels from general Japanese populations, we compared them with the published data of the National Nutrition Survey (NNS) in 1995^1^. The mean intakes of food group and selected nutrients were reported by age-range (every 10 years) and sex, and the mean intakes age-adjusted for the study subjects by sex were used in this study. Because of a slightly different definition of food groups and survey methods, some food groups such as pickled vegetables, beverages, and seasonings and spices were not compared in the two surveys. Mean total intakes of nutrient and food groups were compared with the results from the NNS by dividing the difference between mean intake of our study and the NNS by mean intake of the NNS (percent difference). 

## RESULTS

Tables 1 and 2 show the intake levels of energy and main nutrients by sex and area. The mean energy intake was highest in the Ninohe PHC area, and lowest in the Ishikawa PHC area for both men and women. Mean intakes were significantly different among areas for most of the nutrients except alcohol, retinol, niacin and vitamin C for both sexes, and for total fat, carotene, vitamin B1 and B_2_ for women (p<0.05). Mean intakes were close to the values reported in the NNS among similar age-groups in the same year,^1^ i.e., difference within 10% for all nutrients examined except for sodium and vitamin C in both sexes. After adjustment for energy, the area differences were significant only for total fat, carbohydrate, calcium, sodium, carotene, niacin for both sexes, and for vitamin B_2_ and C for men (p<0.05).

**Table 1.** Nutrient intakes (crude values) assessed with DR by area

Sex Nutrient	Ninohe PHC area (28-days)				Yokote PHC area (28-days)				Saku PHC area (28-days)				Ishikawa PHC area (14-days)				Total				ANOVA^1^	National Nutrition Survey^2^	
						
	Mean	±	SD	Median	Mean	±	SD	Median	Mean	±	SD	Median	Mean	±	SD	Median	Mean	±	SD	Median	p-value	Mean	% difference^3^
Men	(n=24)				(n=28)				(n=23)				(n=27)				(n=102)						
Energy (kcal/day)	2595	±	448	2613	2256	±	297	2277	2549	±	375	2556	2046	±	367	2079	2347	±	430	2327	<0.001	2363	-1
Protein (g/day)	102	±	16	105	91	±	12	91	97	±	14	101	83	±	14	85	93	±	16	93	<0.001	96	-3
Total fat (g/day)	59.4	±	10.6	61.6	53.7	±	8.3	52.3	61.5	±	9.5	62.0	62.8	±	11.8	63.6	59.2	±	10.6	60.5	0.007	61	-2
Carbohydrate (g/day)	370	±	87	344	309	±	59	309	354	±	69	344	246	±	46	257	317	±	81	307	<0.001	323	-2
Alcohol (g/day)	21.8	±	18.0	19.4	23.1	±	16.6	19.8	24.8	±	23.3	14.5	20.8	±	30.1	11.0	22.6	±	22.4	15.8	0.935	---	---
Calcium (mg/day)	720	±	175	695	586	±	116	612	734	±	159	704	482	±	154	474	623	±	181	621	<0.001	627	-1
Phosphorus (mg/day)	1593	±	274	1652	1381	±	184	1406	1541	±	219	1603	1182	±	215	1195	1414	±	273	1402	<0.001	---	---
Iron (mg/day)	14.2	±	2.6	13.9	12.5	±	2.1	11.9	13.6	±	2.6	13.2	11.4	±	2.5	11.4	12.9	±	2.6	12.8	0.001	13.9	-8
Sodium (mg/day)	5650	±	1201	5338	5692	±	930	5670	6049	±	1270	5835	4071	±	756	3996	5334	±	1288	5187	<0.001	6273	15
Potassium (mg/day)	3545	±	720	3548	3130	±	504	3027	3423	±	552	3399	2843	±	644	2673	3218	±	659	3171	<0.001	---	---
Retinol (*µ*g/day)	521	±	437	330	290	±	266	212	480	±	472	371	486	±	631	245	439	±	471	276	0.266	---	---
Carotene (*µ*g/day)	3578	±	1320	3328	2506	±	798	2446	3175	±	874	2972	3905	±	1618	3919	3279	±	1304	2885	<0.001	---	---
Vitamin B_1_ (mg/day)	1.47	±	0.36	1.36	1.19	±	0.18	1.22	1.41	±	0.21	1.45	1.24	±	0.28	1.30	1.32	±	0.29	1.32	0.001	1.34	-2
Vitamin B_2_ (mg/day)	1.76	±	0.38	1.83	1.48	±	0.26	1.48	1.70	±	0.34	1.63	1.32	±	0.31	1.35	1.55	±	0.36	1.51	<0.001	1.59	-2
Niacin (mg/day)	23	±	4	22	22	±	4	22	23	±	3	23	21	±	4	21	22	±	4	22	0.258	---	---
Vitamin C (mg/day)	132	±	45	128	124	±	35	125	139	±	40	134	135	±	46	135	132	±	41	129	0.576	149	-11
Cholesterol (mg/day)	451	±	95	455	414	±	87	402	457	±	102	469	359	±	79	368	418	±	97	404	<0.001	---	---
Women	(n=27)				(n=30)				(n=28)				(n=28)				(n=113)						
Energy (kcal/day)	1914	±	306	1906	1858	±	275	1830	1883	±	287	1924	1625	±	328	1599	1820	±	316	1820	0.002	1918	-5
Protein (g/day)	80.6	±	11.4	79.2	78.8	±	10.5	81.2	76.2	±	12.5	75.9	69.1	±	15.3	68.6	76.2	±	13.1	76.1	0.005	79	-4
Total fat (g/day)	51.0	±	8.4	51.7	51.5	±	7.8	51.0	53.4	±	7.7	52.9	55.9	±	13.9	54.3	52.9	±	9.8	52.3	0.235	54	-3
Carbohydrate (g/day)	279	±	59	272	268	±	51	267	272	±	52	275	209	±	45	200	257	±	58	261	<0.001	273	-6
Alcohol (g/day)	2.6	±	5.0	1.1	1.1	±	1.0	0.8	2.1	±	2.4	1.0	0.7	±	1.2	0.4	1.6	±	2.9	0.8	0.055	---	---
Calcium (mg/day)	674	±	147	670	579	±	110	577	653	±	158	629	497	±	190	476	600	±	166	589	<0.001	603	-1
Phosphorus (mg/day)	1268	±	184	1234	1190	±	161	1236	1215	±	212	1223	1017	±	251	999	1172	±	222	1188	<0.001	---	---
Iron (mg/day)	12.2	±	2.4	11.8	11.7	±	1.8	12.0	11.4	±	2.4	11.0	9.8	±	2.7	9.4	11.3	±	2.5	11.2	0.001	12.3	-9
Sodium (mg/day)	4829	±	945	4573	5290	±	910	5234	4976	±	1047	4717	3472	±	737	3390	4652	±	1143	4507	<0.001	5397	-14
Potassium (mg/day)	3177	±	690	3073	2974	±	451	3015	3033	±	614	3041	2619	±	752	2532	2949	±	657	2986	0.011	---	---
Retinol (*µ*g/day)	475	±	484	337	265	±	266	173	314	±	224	233	435	±	606	164	370	±	425	206	0.201	---	---
Carotene (*µ*g/day)	3322	±	1468	3126	2782	±	944	2658	3109	±	971	2837	3574	±	1505	3746	3188	±	1261	2870	0.103	---	---
Vitamin B_1_ (mg/day)	1.20	±	0.24	1.13	1.10	±	0.17	1.12	1.14	±	0.22	1.09	1.07	±	0.32	1.05	1.12	±	0.24	1.11	0.239	1.18	-5
Vitamin B_2_ (mg/day)	1.51	±	0.28	1.53	1.36	±	0.22	1.39	1.45	±	0.26	1.43	1.21	±	0.39	1.13	1.38	±	0.31	1.39	0.002	1.43	-3
Niacin (mg/day)	17.5	±	3.2	16.5	17.2	±	2.8	17.3	16.9	±	2.9	16.5	16.2	±	4.1	15.7	16.9	±	3.2	16.5	0.501	---	---
Vitamin C (mg/day)	133	±	43	123	139	±	39	129	146	±	46	138	140	±	71	117	140	±	51	130	0.838	160	-13
Cholesterol (mg/day)	358	±	80	349	380	±	88	379	372	±	81	375	313	±	86	307	356	±	87	354	0.016	---	---

^1^ANOVA was used to test for the difference among areas.

^2^Age-adjusted. The National Nutrition Survey in 1995.

^3^Percent difference was calculated by (A-B)/B. A: Mean intake from 4 areas of our study. B: Mean intake from National Nutrition Survey.

**Table 2.** Nutrient intakes (energy density) assessed with DR by area

Sex Nutrient	Ninohe PHC area (28-days)				Ninohe PHC area (28-days)				Saku PHC area (28-days)				Ishikawa PHC area (14-days)				Total				ANOVA^1^
				
	Mean	±	SD	Median	Mean	±	SD	Median	Mean	±	SD	Median	Mean	±	SD	Median	Mean	±	SD	Median	p-value
Men	(n=24)				(n=28)				(n=23)				(n=27)				(n=102)				
Protein (%E)	15.8	±	1.7	15.7	16.2	±	1.5	16.1	15.3	±	1.3	15.1	16.4	±	1.7	16.8	16.0	±	1.6	15.9	0.080
Total fat (%E)	20.9	±	3.4	20.6	21.5	±	2.7	21.2	21.9	±	3.0	21.3	27.8	±	4.3	27.9	23.1	±	4.4	22.3	<0.001
Carbohydrate (%E)	56.6	±	5.3	56.8	54.5	±	5.3	54.5	55.7	±	6.5	56.8	48.4	±	6.7	47.7	53.6	±	6.7	54.0	<0.001
Alcohol (%E)	5.9	±	5.0	5.1	7.3	±	5.1	6.8	6.5	±	5.9	4.7	6.4	±	7.7	4.0	6.6	±	6.0	5.0	0.889
Calcium (mg/1000 kcal)	280	±	63	275	260	±	45	259	290	±	58	286	236	±	65	231	265	±	61	262	0.007
Phosphorus (mg/1000 kcal)	617	±	67	610	614	±	49	600	607	±	52	598	580	±	61	588	604	±	58	597	0.091
Iron (mg/1000 kcal)	5.5	±	0.8	5.5	5.6	±	0.7	5.5	5.3	±	0.7	5.3	5.6	±	1.0	5.4	5.5	±	0.8	5.4	0.616
Sodium (mg/1000 kcal)	2192	±	377	2203	2535	±	349	2430	2387	±	427	2403	2022	±	362	2013	2285	±	423	2328	<0.001
Potassium (mg/1000 kcal)	1381	±	251	1343	1389	±	151	1378	1349	±	156	1336	1396	±	232	1381	1380	±	200	1356	0.855
Retinol (*µ*g/1000 kcal)	200	±	154	143	130	±	118	88	186	±	165	133	237	±	306	99	187	±	202	112	0.263
Carotene (*µ*g/1000 kcal)	1415	±	543	1396	1116	±	322	1110	1253	±	321	1215	1916	±	712	1791	1429	±	588	1270	<0.001
Vitamin B_1_ (mg/1000 kcal)	0.57	±	0.11	0.54	0.53	±	0.05	0.53	0.56	±	0.06	0.57	0.61	±	0.11	0.58	0.57	±	0.09	0.55	0.009
Vitamin B_2_ (mg/1000 kcal)	0.69	±	0.14	0.68	0.66	±	0.09	0.64	0.67	±	0.11	0.66	0.65	±	0.10	0.62	0.66	±	0.11	0.65	0.598
Niacin (mg/1000 kcal)	8.8	±	1.3	8.9	9.6	±	1.3	9.5	8.9	±	0.9	8.7	10.2	±	1.2	10.2	9.4	±	1.3	9.2	<0.001
Vitamin C (mg/1000 kcal)	51.4	±	16.3	52.8	54.7	±	13.0	51.0	54.7	±	12.6	52.4	67.1	±	22.7	63.3	57.2	±	17.6	54.0	0.006
Cholesterol (mg/1000 kcal)	176	±	36	178	184	±	36	172	181	±	43	175	177	±	32	176	180	±	36	175	0.836
Women	(n=27)				(n=30)				(n=28)				(n=28)				(n=113)				
Protein (%E)	17.0	±	1.7	17.2	17.0	±	1.2	17.0	16.2	±	1.4	16.6	17.0	±	1.5	17.0	16.8	±	1.5	16.9	0.111
Total fat (%E)	24.1	±	2.8	24.5	25.1	±	3.4	24.7	25.7	±	2.8	26.2	30.8	±	3.7	31.5	26.4	±	4.1	26.0	<0.001
Carbohydrate (%E)	58.1	±	4.1	57.5	57.4	±	4.2	58.0	57.5	±	3.9	57.0	51.6	±	5.3	51.1	56.1	±	5.1	56.7	<0.001
Alcohol (%E)	1.0	±	2.0	0.4	0.4	±	0.3	0.3	0.7	±	0.8	0.4	0.3	±	0.5	0.1	0.6	±	1.1	0.3	0.087
Calcium (mg/1000 kcal)	354	±	61	349	316	±	65	316	345	±	54	333	302	±	89	279	329	±	71	326	0.018
Phosphorus (mg/1000 kcal)	666	±	58	668	643	±	53	644	645	±	50	647	623	±	77	612	644	±	61	645	0.079
Iron (mg/1000 kcal)	6.4	±	1.0	6.4	6.3	±	0.5	6.3	6.0	±	0.7	6.0	6.0	±	0.9	5.8	6.2	±	0.8	6.2	0.139
Sodium (mg/1000 kcal)	2538	±	388	2465	2861	±	406	2741	2639	±	353	2603	2161	±	342	2166	2555	±	449	2537	<0.001
Potassium (mg/1000 kcal)	1663	±	259	1661	1614	±	236	1595	1603	±	130	1607	1601	±	256	1616	1620	±	224	1608	0.718
Retinol (µg/1000 kcal)	257	±	281	199	141	±	133	96	172	±	136	120	244	±	304	106	202	±	228	114	0.163
Carotene (µg/1000 kcal)	1750	±	747	1674	1524	±	558	1407	1632	±	343	1565	2177	±	714	2077	1767	±	651	1699	0.001
Vitamin B_1_ (mg/1000 kcal)	0.63	±	0.12	0.62	0.59	±	0.07	0.58	0.60	±	0.06	0.60	0.65	±	0.11	0.64	0.62	±	0.09	0.61	0.063
Vitamin B_2_ (mg/1000 kcal)	0.79	±	0.12	0.77	0.74	±	0.11	0.73	0.77	±	0.09	0.76	0.74	±	0.15	0.72	0.76	±	0.12	0.75	0.230
Niacin (mg/1000 kcal)	9.2	±	1.3	8.9	9.3	±	1.0	9.3	9.0	±	1.0	8.8	9.9	±	1.3	10.0	9.4	±	1.2	9.3	0.021
Vitamin C (mg/1000 kcal)	69.3	±	18.2	65.7	75.4	±	21.2	73.5	76.9	±	16.6	75.5	84.2	±	33.5	77.6	76.5	±	23.6	73.3	0.134
Cholesterol (mg/1000 kcal)	189	±	40	183	205	±	41	201	200	±	42	204	193	±	40	199	197	±	41	201	0.473

^1^ANOVA was used to test for the difference among areas.

Tables 3 and 4 show the intake levels of main nutrients by season and sex. A significant seasonal difference was observed in the mean intake of iron, carotene and vitamin C in both men and women, and in the mean intake of carbohydrate, calcium, sodium, and retinol in women (p<0.05) when the crude intakes in the 3 areas were examined. When the values in the 4 areas were examined between summer and winter, a significant seasonal difference was observed in the mean intake of iron and carotene in both sexes, and for calcium, retinol and vitamin B_1_ in women. When the energy-adjustment values in the 3 areas were examined, a significant seasonal difference was observed in the mean intake of protein, iron, potassium, carotene, niacin and vitamin C in men, and in total fat, carbohydrate, calcium, iron, sodium, potassium, retinol, carotene, vitamin B2, vitamin C in women (p<0.05). When the energy-adjusted values in 4 areas were examined between summer and winter, a significant seasonal difference was observed in the mean intake of protein, iron, potassium and retinol in men, and in protein, calcium, iron, potassium, retinol, carotene, vitamin B_1_ and vitamin B_2_ in women.

**Table 3.** Nutrient intakes (crude values) assessed with DR by season

Sex Nutrient	3 areas (Ninohe, Yokote, and Saku PHC areas)																	4 areas								
	
	Winter (7-days)				Spring (7-days)				Summer (7-days)				Autumn (7-days)				Two-way ANOVA^1^	Winter (7-days)				Summer (7-days)				Paired t-test^1^
					
	Mean	±	SD	Median	Mean	±	SD	Median	Mean	±	SD	Median	Mean	±	SD	Median		Mean	±	SD	Median	Mean	±	SD	Median	
Men	(n=75)																	(n=102)								
Energy (kcal/day)	2415	±	413	2393	2447	±	457	2410	2466	±	497	2400	2491	±	449	2473	0.343	2322	±	437	2298	2350	±	505	2286	0.343
Protein (g/day)	97.2	±	18.6	97.5	95.3	±	16.5	97.1	95.5	±	17.5	97.1	97.2	±	16.8	97.4	0.495	94.0	±	18.7	93.8	91.9	±	18.0	91.1	0.495
Total fat (g/day)	58.3	±	14.1	57.4	57.0	±	11.8	55.5	58.1	±	11.7	56.9	58.3	±	13.7	57.8	0.814	59.4	±	13.9	60.0	59.5	±	12.7	57.6	0.814
Carbohydrate (g/day)	332	±	73	332	341	±	82	323	346	±	99	327	351	±	81	347	0.065	311	±	76	309	318	±	100	309	0.065
Alcohol (g/day)	23.1	±	21.1	17.1	23.9	±	21.0	20.5	23.1	±	21.1	21.8	22.6	±	21.7	16.0	0.906	22.3	±	24.7	15.4	22.7	±	23.3	16.6	0.906
Calcium (mg/day)	692	±	194	660	646	±	174	613	667	±	215	655	691	±	209	662	0.093	638	±	209	612	617	±	218	597	0.093
Phosphorus (mg/day)	1491	±	283	1509	1486	±	261	1491	1509	±	301	1466	1506	±	283	1487	0.749	1414	±	304	1425	1418	±	317	1418	0.749
Iron (mg/day)	14.0	±	3.2	13.7	13.2	±	2.7	12.9	13.0	±	3.1	12.8	13.3	±	2.9	12.9	0.004	13.6	±	3.2	13.0	12.4	±	3.1	12.4	0.004
Sodium (mg/day)	5851	±	1472	5604	5607	±	1264	5538	5881	±	1357	5873	5813	±	1231	5719	0.139	5433	±	1483	5210	5349	±	1544	5231	0.139
Potassium (mg/day)	3380	±	712	3260	3281	±	657	3257	3406	±	787	3309	3343	±	718	3299	0.271	3297	±	732	3193	3198	±	817	3099	0.271
Retinol (*µ*g/day)	462	±	555	206	412	±	677	190	359	±	949	183	455	±	977	205	0.866	463	±	581	204	399	±	891	178	0.866
Carotene (*µ*g/day)	3784	±	1612	3671	2962	±	1359	2723	2571	±	1446	2234	2900	±	1472	2375	<0.001	3954	±	1593	3862	2786	±	1721	2332	<0.001
Vitamin B_1_ (mg/day)	1.33	±	0.27	1.33	1.39	±	0.56	1.25	1.34	±	0.37	1.30	1.32	±	0.26	1.32	0.394	1.32	±	0.30	1.33	1.30	±	0.35	1.27	0.394
Vitamin B_2_ (mg/day)	1.66	±	0.40	1.58	1.62	±	0.37	1.56	1.64	±	0.49	1.57	1.63	±	0.45	1.52	0.822	1.57	±	0.42	1.56	1.56	±	0.47	1.51	0.822
Niacin (mg/day)	22.2	±	5.0	21.4	22.5	±	4.5	22.6	22.7	±	5.1	22.7	21.6	±	4.6	21.3	0.289	22.1	±	5.0	21.4	22.0	±	5.1	22.2	0.289
Vitamin C (mg/day)	130	±	41	129	113	±	41	110	127	±	76	120	154	±	60	146	<0.001	127	±	41	124	135	±	76	123	<0.001
Cholesterol (mg/day)	429	±	118	427	426	±	117	418	449	±	137	421	451	±	118	450	0.469	408	±	118	396	428	±	133	404	0.469
Women	(n=85)																	(n=113)								
Energy (kcal/day)	1861	±	311	1827	1892	±	365	1820	1883	±	352	1843	1900	±	311	1874	0.657	1809	±	322	1800	1811	±	379	1797	0.657
Protein (g/day)	79.1	±	15.4	77.6	78.8	±	14.3	76.2	77.4	±	13.7	78.2	78.7	±	129	78.4	0.634	77.2	±	15.6	74.3	74.8	±	15.4	74.5	0.634
Total fat (g/day)	52.5	±	11.4	51.9	52.0	±	11.3	51.2	52.9	±	11.0	52.3	50.4	±	9.2	50.6	0.187	53.7	±	12.2	52.4	53.3	±	13.0	52.3	0.187
Carbohydrate (g/day)	266	±	51	265	273	±	65	263	271	±	65	272	281	±	60	273	0.041	253	±	55	253	255	±	67	252	0.041
Alcohol (g/day)	1.8	±	4.1	0.8	1.8	±	3.2	0.7	2.0	±	3.3	0.7	2.0	±	3.5	0.9	0.680	1.5	±	3.6	0.7	1.7	±	3.1	0.6	0.680
Calcium (mg/day)	662	±	179	633	616	±	159	616	623	±	174	609	632	±	181	607	0.041	631	±	201	617	582	±	191	561	0.041
Phosphorus (mg/day)	1228	±	238	1198	1230	±	225	1219	1220	±	218	1221	1214	±	212	1225	0.882	1187	±	254	1152	1159	±	256	1183	0.882
Iron (mg/day)	12.6	±	2.9	12.2	11.6	±	2.6	11.3	11.3	±	2.3	10.9	11.6	±	2.4	11.4	<0.001	12.1	±	3.0	11.6	10.7	±	2.6	10.6	<0.001
Sodium (mg/day)	5226	±	1299	5024	4799	±	1147	4625	5103	±	1091	4986	5033	±	1168	4894	0.003	4819	±	1377	4595	4672	±	1317	4641	0.003
Potassium (mg/day)	3119	±	772	2993	2967	±	651	2861	3114	±	696	2995	3032	±	645	2947	0.050	3051	±	784	2966	2936	±	782	2951	0.050
Retinol (*µ*g/day)	503	±	931	173	326	±	483	167	277	±	357	176	285	±	364	163	0.039	501	±	876	171	302	±	446	168	0.039
Carotene (*µ*g/day)	3767	±	1613	3478	2910	±	1380	2754	2768	±	1418	2356	2799	±	1457	2354	<0.001	3828	±	1617	3655	2859	±	1580	2456	<0.001
VitaminB_1_ (mg/day)	1.15	±	0.29	1.08	1.16	±	0.34	1.09	1.14	±	0.24	1.11	1.12	±	0.23	1.09	0.627	1.15	±	0.31	1.08	1.10	±	0.27	1.11	0.627
Vitamin B_2_ (mg/day)	1.47	±	0.34	1.41	1.43	±	0.30	1.42	1.43	±	0.31	1.42	1.41	±	0.30	1.34	0.261	1.42	±	0.37	1.41	1.36	±	0.36	1.36	0.261
Niacin (mg/day)	17.1	±	4.0	16.5	17.5	±	4.3	16.4	17.2	±	3.6	16.9	17.0	±	3.2	17.0	0.729	17.1	±	4.1	16.5	16.7	±	4.0	16.7	0.729
Vitamin C (mg/day)	145	±	51	139	120	±	48	106	131	±	57	119	163	±	63	158	<0.001	136	±	50	131	140	±	78	119	<0.001
Cholesterol (mg/day)	367	±	109	356	360	±	108	350	379	±	121	372	376	±	105	376	0.689	355	±	108	341	361	±	121	355	0.689

^1^P-value for seasonal difference after adjusting for area difference.

**Table 4.** Nutrient intakes (energy density) assessed with DR by season

Sex Nutrient	3 areas (Ninohe, Yokote, and Saku PHC areas)																	4 areas								
	
	Winter (7-days)				Spring (7-days)				Summer (7-days)				Autumn (7-days)				Two-way ANOVA^1^	Winter (7-days)				Summer (7-days)				Paired t-test^1^
					
	Mean	±	SD	Median	Mean	±	SD	Median	Mean	±	SD	Median	Mean	±	SD	Median		Mean	±	SD	Median	Mean	±	SD	Median	
Men	(n=75)																	(n=102)								
Protein (%E)	16.2	±	1.9	16.0	15.7	±	1.8	15.5	15.7	±	1.9	15.4	15.7	±	1.8	15.5	0.048	16.3	±	1.9	16.3	15.8	±	2.0	15.5	0.014
Total fat (%E)	21.8	±	4.0	21.5	21.2	±	3.7	21.2	21.6	±	3.9	21.2	21.2	±	4.0	21.1	0.483	23.3	±	4.9	22.6	23.3	±	5.2	23.2	0.954
Carbohydrate (%E)	54.8	±	6.2	54.2	55.6	±	6.0	55.5	55.5	±	7.1	55.9	56.2	±	6.4	56.5	0.167	53.3	±	6.8	52.9	53.4	±	7.9	54.7	0.839
Alcohol (%E)	6.7	±	5.9	5.7	6.7	±	5.7	5.5	6.6	±	5.8	5.4	6.3	±	5.9	4.4	0.796	6.5	±	6.5	4.6	6.7	±	6.5	4.9	0.769
Calcium (mg/1000 kcal)	286	±	60	279	267	±	65	251	273	±	79	262	279	±	75	271	0.094	273	±	68	268	263	±	79	259	0.160
Phosphorus (mg/1000 kcal)	619	±	65	611	611	±	65	608	616	±	74	609	607	±	69	594	0.389	609	±	70	606	606	±	75	603	0.698
Iron (mg/1000 kcal)	5.8	±	1.0	5.6	5.4	±	1.0	5.4	5.3	±	0.9	5.1	5.4	±	0.9	5.4	<0.001	5.9	±	1.0	5.6	5.3	±	1.0	5.2	<0.001
Sodium (mg/1000 kcal)	2440	±	507	2365	2321	±	483	2254	2423	±	498	2417	2364	±	459	2313	0.071	2358	±	509	2348	2291	±	524	2288	0.165
Potassium (mg/1000 kcal)	1407	±	219	1396	1355	±	224	1329	1392	±	245	1372	1351	±	210	1350	0.036	1430	±	232	1403	1370	±	251	1322	0.010
Retinal (*µ*g/1000 kcal)	191	±	220	86	170	±	280	74	140	±	328	76	178	±	346	79	0.783	200	±	247	84	170	±	337	77	0.433
Carotene (*µ*g/1000 kcal)	1579	±	645	1510	1227	±	544	1224	1048	±	554	877	1182	±	613	1029	<0.001	1736	±	705	1706	1206	±	717	1031	<0.001
Vitamin B_1_ (mg/1000 kcal)	0.6	±	0.1	0.5	0.6	±	0.2	0.5	0.5	±	0.1	0.5	0.5	±	0.1	0.5	0.310	0.6	±	0.1	0.6	0.6	±	0.1	0.5	0.395
Vitamin B_2_ (mg/1000 kcal)	0.7	±	0.1	0.7	0.7	±	0.1	0.6	0.7	±	0.2	0.6	0.7	±	0.1	0.6	0.285	0.7	±	0.1	0.6	0.7	±	0.2	0.6	0.493
Niacin (mg/1000 kcal)	9.3	±	1.6	9.2	9.3	±	1.7	9.2	9.3	±	1.8	9.4	8.7	±	1.4	8.6	0.011	9.6	±	1.6	9.4	9.5	±	1.8	9.7	0.658
Vitamin C (mg/1000 kcal)	54.6	±	16.1	53.7	46.9	±	16.7	43.3	51.0	±	22.9	47.1	62.0	±	22.6	58.9	<0.001	55.0	±	15.7	53.7	58.3	±	31.2	53.1	0.241
Cholesterol (mg/1000 kcal)	180	±	48	183	176	±	47	169	186	±	56	179	183	±	45	184	0.654	177	±	47	183	185	±	52	182	0.275
Women	(n=85)																	(n=113)								
Protein (%E)	17.1	±	3.7	16.6	16.8	±	1.8	16.5	16.6	±	2.0	16.2	16.6	±	1.7	16.6	0.106	17.1	±	1.9	17.3	16.6	±	1.9	16.4	0.014
Total fat (%E)	25.7	±	6.4	25.2	24.9	±	3.9	25.0	25.5	±	4.1	25.8	24.1	±	4.1	24.1	0.005	26.9	±	4.7	26.7	26.7	±	4.7	26.7	0.683
Carbohydrate (%E)	56.9	±	9.0	56.5	57.4	±	4.9	57.4	57.3	±	5.2	57.4	58.8	±	5.1	58.8	0.005	55.7	±	5.7	56.3	56.0	±	5.9	56.5	0.506
Alcohol (%E)	0.7	±	1.6	0.3	0.7	±	1.2	0.3	0.7	±	1.2	0.3	0.7	±	1.3	0.4	0.702	0.5	±	1.3	0.2	0.6	±	1.2	0.2	0.279
Calcium (mg/1000 kcal)	355	±	73	351	329	±	75	333	334	±	84	330	334	±	87	315	0.023	347	±	90	345	322	±	85	315	0.004
Phosphorus (mg/1000 kcal)	661	±	68	655	654	±	71	655	652	±	68	654	641	±	70	636	0.098	655	±	77	647	641	±	71	642	0.053
Iron (mg/1000 kcal)	6.7	±	1.0	6.7	6.2	±	0.9	6.1	6.0	±	1.0	5.8	6.1	±	1.0	5.9	<0.001	6.7	±	1.1	6.7	5.9	±	1.0	5.8	<0.001
Sodium (mg/1000 kcal)	2807	±	505	2752	2552	±	485	2457	2746	±	531	2739	2664	±	534	2608	<0.001	2656	±	553	2647	2589	±	590	2593	0.219
Potassium (mg/1000 kcal)	1672	±	277	1598	1580	±	266	1568	1665	±	279	1616	1598	±	232	1573	0.001	1683	±	297	1616	1620	±	279	1594	0.022
Retinol (*µ*g/1000 kcal)	280	±	549	90	172	±	253	94	154	±	228	92	151	±	193	91	0.042	279	±	508	93	171	±	261	92	0.033
Carotene (*µ*g/1000 kcal)	2013	±	753	1935	1573	±	777	1397	1486	±	728	1353	1477	±	715	1321	<0.001	2112	±	795	2047	1593	±	814	1433	<0.001
Vitamin B_1_ (mg/1000 kcal)	0.62	±	0.13	0.61	0.62	±	0.16	0.59	0.61	±	0.09	0.60	0.59	±	0.09	0.58	0.220	0.64	±	0.14	0.62	0.61	±	0.10	0.60	0.026
Vitamin B_2_ (mg/1000 kcal)	0.80	±	0.15	0.78	0.76	±	0.13	0.74	0.77	±	0.15	0.77	0.75	±	0.14	0.73	0.038	0.79	±	0.16	0.78	0.76	±	0.15	0.76	0.057
Niacin (mg/1000 kcal)	9.2	±	1.5	9.2	9.3	±	1.9	9.0	9.2	±	1.5	9.1	9.0	±	1.3	8.9	0.421	9.5	±	1.6	9.3	9.3	±	1.7	9.1	0.351
Vitamin C (mg/1000 kcal)	77.5	±	22.8	73.7	63.6	±	24.1	56.1	69.9	±	28.5	66.9	85.8	±	30.8	82.1	<0.001	75.0	±	22.6	73.7	77.6	±	40.1	70.2	0.405
Cholesterol (mg/1000 kcal)	199	±	55	191	192	±	51	191	203	±	60	197	199	±	51	202	0.585	198	±	57	190	201	±	57	194	0.745

^1^P-value for seasonal difference after adjusting for area difference.

Tables 5 and 6 show the intake levels of food groups by sex and area. Mean intakes were significantly different among the 4 areas in most of the food groups except for potatoes and starches, and alcoholic and nonalcoholic beverages in both sexes, algae in men, and green and yellow vegetables, and milks in women (p<0.05). The difference between the observed intakes and the reported ones from the NNS was 040% to 60% larger than that observed for nutrients. After the adjustment for energy, the area differences were significant for most of the food groups with the exception of cereals, sugar and sweeteners, eggs, vegetables. algae and non-alcoholic beverages in men, and except for cereals, potatoes and starches, eggs, milks, vegetables, green and yellow vegetables, and alcoholic beverages in women (p<0.05).

**Table 5.** Food group intakes (g/day) assessed with DR by area.

Sex Food group	Ninohe PHC area (28-days)				Yokote PHC area (28-days)				Saku PHC area (28-days)				Ishikawa PHC area (14-days)				Total				ANOVA^2^ p-value	National Nutrition Survey^3^	
					
	Mean	±	SD	Median	Mean	±	SD	Median	Mean	±	SD	Median	Mean	±	SD	Median	Mean	±	SD	Median		Mean	% difference^3^
Men	(n=24)				(n=28)				(n=23)				(n=27)				(n=102)						
Cereals	360	±	97	338	288	±	54	291	355	±	96	360	262	±	51	270	313	±	86	296	<0.001	295	6
Potatoes and starches	48	±	18	47	41	±	13	37	57	±	33	53	59	±	35	53	51	±	27	47	0.054	69	-26
Sugar and sweeteners	8	±	7	8	8	±	5	7	10	±	5	9	6	±	5	4	8	±	6	7	0.036	10	-19
Confectioneries	23	±	18	18	38	±	33	27	37	±	27	27	15	±	17	9	28	±	26	21	0.003	20	40
Fats and oils	10	±	3	10	9	±	3	9	10	±	3	11	15	±	5	15	11	±	4	10	<0.001	18	-37
Nuts and seeds	3	±	2	2	2	±	2	1	4	±	6	2	0	±	1	0	2	±	3	1	<0.001	2	11
Pulses	110	±	37	114	96	±	29	93	66	±	21	71	77	±	40	72	87	±	36	82	<0.001	76	14
Fish and shellfish	151	±	44	146	153	±	44	155	137	±	36	135	102	±	41	88	136	±	46	137	<0.001	107	27
Meats	67	±	24	63	58	±	19	58	72	±	28	62	107	±	34	100	76	±	32	69	<0.001	90	-15
Eggs	41	±	12	41	37	±	15	32	46	±	15	45	35	±	12	33	39	±	14	38	0.016	45	-13
Milks	159	±	144	140	99	±	80	75	172	±	100	157	76	±	84	50	124	±	110	109	0.003	132	-7
Vegetables	344	±	93	347	292	±	83	306	344	±	81	347	286	±	87	275	314	±	89	307	0.018	291	8
Green & yellow	117	±	59	118	88	±	33	86	123	±	43	119	110	±	49	99	108	±	48	100	0.041	99	10
Pickled^1^	29	±	23	27	38	±	23	31	68	±	25	76	6	±	5	5	34	±	30	25	<0.001	---	---
Fruits	150	±	106	111	135	±	88	119	137	±	66	112	64	±	38	67	120	±	85	95	0.001	105	14
Fungi	7	±	5	7	10	±	6	9	12	±	8	9	6	±	5	5	9	±	6	7	0.003	13	-34
Algae	7	±	4	7	9	±	8	7	6	±	4	5	7	±	6	6	8	±	6	6	0.251	6	36
Alcoholic beverages	273	±	246	244	409	±	309	371	271	±	221	255	272	±	294	171	309	±	276	260	0.172	---	---
Non-alcoholic beverages	293	±	155	251	261	±	152	224	315	±	110	326	229	±	102	214	272	±	134	263	0.113	---	---
Seasonings and spices	44	±	10	45	34	±	8	34	41	±	9	40	26	±	5	26	36	±	10	35	<0.001	---	---
Women	(n=27)				(n=30)				(n=28)				(n=28)				(n=113)						
Cereals	237	±	50	225	220	±	52	219	231	±	44	230	195	±	40	190	220	±	49	217	0.006	234	-6
Potatoes and starches	45	±	20	39	49	±	19	44	50	±	23	47	50	±	29	45	49	±	23	44	0.809	67	-27
Sugar and sweeteners	7	±	5	6	8	±	3	7	11	±	6	9	6	±	3	5	8	±	5	7	<0.001	10	-19
Confectioneries	41	±	25	34	64	±	38	60	53	±	26	53	36	±	30	23	49	±	32	46	0.004	28	72
Fats and oils	9	±	2	8	8	±	3	8	9	±	3	9	13	±	5	12	10	±	4	9	<0.001	16	-40
Nuts and seeds	4	±	8	2	2	±	2	1	4	±	3	3	0	±	1	0	3	±	5	1	0.002	2	17
Pulses	93	±	31	97	87	±	19	84	59	±	16	58	57	±	27	61	74	±	29	71	<0.001	69	8
Fish and shellfish	116	±	31	117	127	±	27	128	104	±	27	99	78	±	27	77	106	±	33	102	<0.001	88	20
Meats	52	±	19	48	52	±	16	52	51	±	19	48	85	±	40	77	60	±	29	52	<0.001	67	-11
Eggs	31	±	12	32	34	±	12	32	39	±	13	37	28	±	10	29	33	±	12	32	0.010	39	-15
Milks	174	±	103	175	124	±	78	135	173	±	80	163	139	±	106	102	152	±	94	155	0.100	135	12
Vegetables	315	±	90	294	314	±	91	304	317	±	78	303	256	±	93	255	300	±	91	289	0.026	281	7
Green & yellow	113	±	53	103	103	±	40	96	126	±	47	111	104	±	53	89	111	±	48	103	0.244	99	12
Pickled^1^	23	±	15	22	41	±	22	35	50	±	28	41	5	±	4	4	30	±	26	22	<0.001	---	---
Fruits	192	±	98	211	157	±	72	153	181	±	71	172	96	±	62	78	156	±	84	144	<0.001	131	19
Fungi	7	±	5	6	9	±	6	9	11	±	6	8	5	±	4	4	8	±	6	7	0.001	12	-33
Algae	6	±	4	5	10	±	7	8	6	±	4	5	6	±	4	4	7	±	5	5	0.021	5	31
Alcoholic beverages	38	±	63	13	17	±	22	8	29	±	39	9	13	±	29	4	24	±	41	8	0.107	---	---
Non-alcoholic beverages	274	±	121	238	256	±	122	228	302	±	108	286	286	±	105	289	279	±	114	270	0.469	---	---
Seasonings and spices	39	±	10	37	33	±	7	32	37	±	8	37	23	±	6	22	33	±	10	33	<0.001	---	---

^1^Pickled plum (umeboshi) was included in pickled vegetables, and not in total vegetables but rather in fruits.

^2^ANOVA was used to test for the difference among areas.

^3^Age-adjusted values. The National Nutrition Survey in 1995.

^4^Percent difference was calculated by (A-B)/B. A: Mean intake from 4 areas of our study. B: Mean intake from National Nutrition Survey.

**Table 6.** Food group intakes in energy density (g/1000 kcal) assessed with DR by area

Sex Food group	Ninohe PHC area (28-days)				Yokote PHC area (28-days)				Saku PHC area (28-days)				Ishikawa PHC area (14-days)				Total				ANOVA^2^ p-value
				
	Mean	±	SD	Median	Mean	±	SD	Median	Mean	±	SD	Median	Mean	±	SD	Median	Mean	±	SD	Median	
Men	(n=24)				(n=28)				(n=23)				(n=27)				(n=102)				
Cereals	137	±	21	143	128	±	18	128	139	±	27	138	130	±	23	131	133	±	23	133	0.210
Potatoes	18	±	7	20	18	±	6	17	22	±	11	20	29	±	14	27	22	±	11	21	0.001
Sugar and sweeteners	3	±	2	3	3	±	2	3	4	±	2	3	3	±	2	2	3	±	2	3	0.191
Confectioneries	9	±	7	7	16	±	13	12	14	±	9	12	7	±	8	5	12	±	10	9	0.003
Fats and oils	4	±	1	4	4	±	1	4	4	±	1	4	8	±	3	7	5	±	3	4	<0.001
Nuts and seeds	1	±	1	1	1	±	1	1	2	±	2	1	0	±	0	0	1	±	1	0	<0.001
Pulses	42	±	14	46	43	±	13	41	25	±	6	25	38	±	21	36	38	±	16	35	<0.001
Fish and shellfish	59	±	17	56	68	±	19	66	54	±	12	52	51	±	21	47	58	±	19	54	0.003
Meats	26	±	10	23	26	±	9	25	28	±	10	27	52	±	11	51	33	±	15	30	0.000
Eggs	16	±	5	16	16	±	7	14	18	±	7	17	17	±	6	16	17	±	6	16	0.624
Milks	61	±	57	58	44	±	35	38	69	±	41	65	36	±	38	26	51	±	44	42	0.029
Vegetables	135	±	36	135	128	±	27	124	136	±	28	128	140	±	37	133	135	±	32	130	0.588
Green & yellow	46	±	25	47	39	±	13	38	48	±	14	45	53	±	19	52	46	±	19	45	0.040
Pickled^1^	11	±	9	9	16	±	9	14	27	±	10	26	3	±	2	2	14	±	12	10	<0.001
Fruits	58	±	41	48	59	±	35	55	53	±	23	46	31	±	18	32	50	±	32	41	0.004
Fungi	3	±	2	3	4	±	3	4	5	±	3	3	3	±	2	2	4	±	2	3	0.016
Algae	3	±	2	3	4	±	4	3	2	±	2	2	4	±	3	3	3	±	3	3	0.141
Alcoholic beverages	103	±	89	86	183	±	137	155	105	±	83	88	127	±	137	82	132	±	119	111	0.046
Non-alcoholic beverages	114	±	59	112	112	±	61	100	125	±	44	121	113	±	48	116	116	±	53	113	0.818
Seasonings and spices	17	±	3	16	15	±	3	15	16	±	3	16	13	±	3	13	15	±	4	15	<0.001
Women	(n=27)				(n=30)				(n=28)				(n=28)				(n=113)				
Cereals	124	±	17	124	118	±	20	114	123	±	17	122	122	±	25	120	122	±	20	120	0.661
Potatos and starches	24	±	10	20	26	±	10	26	26	±	9	25	31	±	16	27	27	±	12	25	0.159
Sugar and sweeteners	4	±	2	3	4	±	2	4	6	±	3	4	4	±	2	3	4	±	2	4	0.004
Confectioneries	21	±	12	20	34	±	17	31	28	±	12	25	22	±	17	19	26	±	15	24	0.005
Fats and oils	5	±	1	4	5	±	1	5	5	±	1	5	8	±	3	8	5	±	2	5	<0.001
Nuts and seeds	2	±	4	1	1	±	1	1	2	±	2	2	0	±	0	0	1	±	2	1	0.003
Pulses	50	±	17	47	47	±	9	46	31	±	6	31	35	±	14	33	41	±	14	39	<0.001
Fish and shellfish	61	±	15	56	68	±	11	67	55	±	12	55	48	±	17	48	58	±	16	60	<0.001
Meats	28	±	11	26	28	±	10	25	27	±	10	26	51	±	17	48	34	±	16	30	<0.001
Eggs	17	±	7	16	19	±	7	18	21	±	8	21	18	±	6	16	18	±	7	18	0.160
Milks	90	±	51	86	69	±	45	69	92	±	39	98	83	±	58	68	83	±	49	79	0.270
Vegetables	165	±	40	171	170	±	52	159	168	±	28	169	157	±	42	155	165	±	42	160	0.628
Green & yellow	59	±	27	57	57	±	24	53	66	±	18	65	63	±	25	64	61	±	24	59	0.475
Pickled^1^	12	±	7	11	22	±	12	19	26	±	14	23	3	±	2	2	16	±	13	12	<0.001
Fruits	98	±	45	96	85	±	37	80	95	±	29	88	58	±	35	50	84	±	40	81	<0.001
Fungi	4	±	3	3	5	±	3	5	6	±	3	5	3	±	2	2	4	±	3	4	0.003
Algae	3	±	2	3	5	±	4	4	3	±	2	2	4	±	3	3	4	±	3	3	0.037
Alcoholic beverages	19	±	32	6	9	±	11	4	15	±	19	6	8	±	19	2	13	±	21	4	0.152
Non-alcoholic beverages	144	±	61	127	137	±	61	130	163	±	55	171	180	±	67	183	156	±	63	157	0.041
Seasonings and spices	20	±	4	21	18	±	4	18	20	±	3	19	14	±	4	14	18	±	4	18	<0.001

^1^Pickled plum (umeboshi) was included in pickled vegetables, and not in total vegetables but rather in fruits.

^2^ANOVA was used to test for the difference among areas.

Tables 7 and 8 show the intake levels of food groups by season and sex. A significant seasonal difference was observed in the mean intake of potatoes and starches, fats and oils, vegetables, green and yellow vegetables, pickled vegetables, fruits, fungi, algae, alcoholic beverages in men, and in the mean intake of potatoes and starches, fats and oils, pulses, milks, vegetables, green and yellow vegetables, pickled vegetables, fruits and fungi among women (p<0.05), when the crude values in the 3 areas were examined. In the 4 areas, a significant seasonal difference was observed between summer and winter in the mean intake of potatoes and starches, sugar and sweeteners, nuts and seeds, milks, vegetables, pickled vegetables, fruits, fungi, algae, and alcoholic beverages in men, and in the mean intake of potatoes and starches, fats and oils, nuts and seeds, pulses, milks, vegetables, green and yellow vegetables, pickled vegetables, fruits and fungi in women (p<0.05). This seasonal difference in 3 areas was consistent after energy adjustment for most food groups except confectioneries in men (p<0.05). The seasonal difference in 4 areas was also consistent for most food groups aside from milks and algae in men, and alcoholic beverages in women (p<0.05).

**Table 7.** Food group intakes (g/day) assessed with DR by season

Sex Food group	3 areas (Ninohe, Yokote, and Saku PHC)																	4 areas (3 areas and Ishikawa PHC)								
	
	Winter (7-days)				Spring (7-days)				Summer (7-days)				Autumn (7-days)				Two-way ANOVA^2^	Winter (7-days)				Summer (7-days)				Paired t-test p-value
					
	Mean	±	SD	Median	Mean	±	SD	Median	Mean	±	SD	Median	Mean	±	SD	Median		Mean	±	SD	Median	Mean	±	SD	Median	
Men	(n=75)				(n=75)				(n=75)				(n=75)					(n=75)				(n=102)				
Cereals	324	±	92	313	331	±	97	316	332	±	113	318	338	±	94	331	0.469	309	±	88	294	312	±	106	295	0.735
Potatoes and starches	55	±	34	50	37	±	27	32	39	±	29	35	63	±	35	58	<0.001	60	±	37	56	39	±	32	35	<0.001
Sugar and sweeteners	10	±	8	8	8	±	6	8	8	±	7	7	8	±	7	7	0.158	9	±	8	7	7	±	7	6	0.013
Confectioneries	34	±	39	24	38	±	33	29	29	±	33	19	30	±	29	22	0.051	29	±	36	19	26	±	32	12	0.302
Fats and oils	9	±	4	9	10	±	5	9	11	±	4	11	9	±	5	9	0.007	11	±	6	10	12	±	5	11	0.053
Nuts and seeds	3	±	6	1	2	±	3	1	2	±	3	0	4	±	9	1	0.053	3	±	6	1	1	±	3	0	0.013
Pulses	94	±	44	84	94	±	47	86	85	±	39	83	91	±	44	86	0.306	89	±	44	81	83	±	42	78	0.172
Fish and shellfish	151	±	59	141	143	±	52	143	143	±	50	132	154	±	48	144	0.190	139	±	59	135	131	±	53	121	0.149
Meats	66	±	36	65	62	±	27	56	67	±	33	61	66	±	31	63	0.503	77	±	41	68	78	±	40	69	0.779
Eggs	40	±	16	39	41	±	19	38	42	±	19	41	42	±	19	39	0.819	38	±	16	38	40	±	18	39	0.310
Milks	131	±	106	121	141	±	125	94	156	±	145	138	134	±	133	94	0.114	114	±	105	84	138	±	137	119	0.023
Vegetables	302	±	97	286	310	±	92	314	388	±	157	352	299	±	98	282	<0.001	301	±	95	282	358	±	152	334	<0.001
Green & yellow	106	±	49	101	111	±	57	101	132	±	91	102	82	±	46	70	<0.001	112	±	49	106	121	±	85	93	0.130
Pickled^1^	38	±	32	30	29	±	27	19	62	±	53	54	48	±	36	42	<0.001	30	±	31	20	47	±	52	30	<0.001
Fruits	119	±	84	102	109	±	95	90	166	±	146	134	167	±	129	130	<0.001	100	±	82	87	143	±	135	112	0.001
Fungi	12	±	10	9	6	±	7	4	8	±	10	5	12	±	12	9	<0.001	10	±	9	8	7	±	9	4	0.003
Algae	6	±	5	5	8	±	8	5	11	±	17	5	6	±	8	4	0.031	7	±	6	5	10	±	15	5	0.049
Alcoholic beverages	280	±	295	238	361	±	345	244	387	±	334	297	264	±	273	197	<0.001	266	±	296	206	368	±	334	274	<0.001
Non-alcoholic beverages	287	±	153	274	287	±	151	267	300	±	176	276	276	±	154	274	0.382	266	±	148	257	287	±	164	274	0.114
Seasonings and spices	40	±	13	38	40	±	13	39	39	±	13	38	39	±	11	38	0.563	37	±	13	34	35	±	13	32	0.120
Women	(n=85)				(n=85)				(n=85)				(n=85)					(n=113)				(n=113)				
Cereals	220	±	50	217	234	±	59	232	228	±	66	225	233	±	66	226	0.157	214	±	50	209	220	±	63	213	0.314
Potatoes and starches	55	±	32	49	36	±	25	31	39	±	24	38	61	±	37	54	<0.001	57	±	33	50	39	±	26	35	<0.001
Sugar and sweeteners	9	±	7	9	9	±	7	8	8	±	6	6	8	±	5	7	0.102	9	±	7	7	8	±	6	6	0.069
Confectioneries	55	±	38	51	56	±	43	47	48	±	39	42	53	±	36	45	0.214	50	±	37	46	46	±	38	40	0.141
Fats and oils	8	±	4	8	8	±	4	8	10	±	5	9	8	±	4	8	0.002	9	±	5	9	11	±	5	10	0.018
Nuts and seeds	3	±	5	1	2	±	4	1	2	±	2	1	6	±	18	2	0.069	3	±	4	1	1	±	2	0	0.001
Pulses	85	±	38	79	82	±	38	76	73	±	33	65	80	±	33	72	0.028	78	±	38	75	69	±	34	63	0.028
Fish and shellfish	116	±	41	108	111	±	41	106	113	±	40	107	123	±	41	122	0.068	107	±	43	101	103	±	41	101	0.471
Meats	52	±	32	46	52	±	22	53	52	±	24	50	50	±	21	46	0.780	61	±	38	52	59	±	33	54	0.521
Eggs	33	±	15	33	35	±	17	34	35	±	20	33	36	±	17	34	0.659	32	±	15	32	34	±	18	33	0.305
Milks	140	±	93	130	166	±	102	169	178	±	110	187	140	±	104	146	<0.001	139	±	99	128	169	±	112	173	0.001
Vegetables	294	±	110	287	298	±	94	287	391	±	134	365	278	±	98	259	<0.001	288	±	106	275	354	±	144	335	<0.001
Green & yellow	112	±	60	107	112	±	54	106	152	±	87	137	79	±	48	64	<0.001	114	±	59	107	136	±	86	117	0.003
Pickled^1^	37	±	32	29	24	±	23	17	54	±	41	38	38	±	29	33	<0.001	29	±	31	17	42	±	42	31	<0.001
Fruits	160	±	93	141	141	±	96	132	193	±	133	167	209	±	100	201	<0.001	136	±	95	114	177	±	128	140	0.001
Fungi	10	±	9	9	6	±	6	4	8	±	8	5	13	±	12	9	<0.001	9	±	8	7	7	±	8	4	0.040
Algae	6	±	5	4	8	±	11	4	10	±	16	4	6	±	7	3	0.069	6	±	6	4	9	±	14	4	0.052
Alcoholic beverages	23	±	53	7	28	±	48	6	33	±	56	7	25	±	51	8	0.209	---				---			---	---
Non-alcoholic beverages	278	±	133	257	280	±	148	243	287	±	155	275	263	±	120	264	0.294	279	±	126	260	288	±	149	279	0.497
Seasonings and spices	36	±	12	35	37	±	13	36	36	±	12	35	35	±	10	34	0.401	33	±	12	31	32	±	13	29	0.359

^1^Pickled plum (umeboshi) was included in pickled vegetables, and not in total vegetables but rather in fruits.

^2^P-value for seasonal difference after adjusting for area difference.

**Table 8.** Food group intakes (g/1000 kcal) assessed with DR by season

Sex Food group	3 areas (Ninohe, Yokote, and Saku PHC areas)																	4 areas (3 areas and Ishikawa PHC area)								
	
	Winter (7-days)				Spring (7-days)				Summer (7-days)				Autumn (7-days)				Two-way ANOVA^2^	Winter (7-days)				Summer (7-days)				Paired t-test p-value
					
	Mean	±	SD	Median	Mean	±	SD	Median	Mean	±	SD	Median	Mean	±	SD	Median		Mean	±	SD	Median	Mean	±	SD	Median	
Men	(n=75)				(n=75)				(n=75)				(n=75)					(n=75)				(n=102)				
Cereals	134	±	28	131	135	±	26	136	134	±	30	127	135	±	24	133	0.936	309	±	88	294	132	±	29	127	0.622
Potatoes and starches	23	±	13	21	15	±	10	14	16	±	12	14	25	±	13	23	<0.001	60	±	37	56	17	±	13	15	<0.001
Sugar and sweeteners	4	±	3	3	3	±	2	3	3	±	3	3	3	±	3	3	0.098	9	±	8	7	3	±	2	2	0.002
Confectioneries	14	±	15	10	15	±	13	12	11	±	12	7	12	±	11	9	0.013	29	±	36	19	10	±	12	5	0.208
Fats and oils	4	±	2	4	4	±	2	4	5	±	2	5	4	±	2	4	0.002	11	±	6	10	5	±	3	5	0.056
Nuts and seeds	1	±	3	0	1	±	1	0	1	±	1	0	2	±	3	0	0.050	3	±	6	1	1	±	1	0	0.011
Pulses	39	±	17	34	39	±	19	35	35	±	17	33	37	±	17	32	0.212	89	±	44	81	36	±	20	33	0.292
Fish and shellfish	63	±	24	56	59	±	22	56	59	±	21	57	63	±	20	58	0.253	139	±	59	135	57	±	22	55	0.101
Meats	28	±	14	26	25	±	11	26	27	±	14	26	27	±	11	25	0.523	77	±	41	68	34	±	18	31	0.777
Eggs	17	±	7	16	17	±	9	16	18	±	9	16	17	±	8	15	0.912	38	±	16	38	18	±	8	16	0.305
Milks	55	±	44	43	58	±	50	42	63	±	59	55	53	±	52	40	0.131	114	±	105	84	58	±	57	51	0.030
Vegetables	125	±	35	122	128	±	38	128	157	±	55	150	121	±	34	116	<0.001	301	±	95	282	152	±	53	146	<0.001
Green & yellow	44	±	20	43	46	±	24	43	54	±	35	46	33	±	18	28	<0.001	112	±	49	106	51	±	32	42	0.310
Pickled^1^	16	±	13	14	12	±	11	8	25	±	21	22	19	±	13	17	<0.001	30	±	31	20	19	±	21	14	<0.001
Fruits	50	±	36	40	44	±	36	37	65	±	49	56	67	±	51	52	<0.001	100	±	82	87	58	±	47	45	0.001
Fungi	5	±	4	4	3	±	3	2	3	±	4	2	5	±	5	3	<0.001	10	±	9	8	3	±	3	2	0.002
Algae	3	±	2	2	3	±	4	2	5	±	8	2	3	±	4	1	0.043	7	±	6	5	4	±	7	2	0.072
Alcoholic beverages	120	±	126	91	149	±	146	107	160	±	141	126	104	±	104	74	<0.001	266	±	296	206	157	±	148	127	<0.001
Non-alcoholic beverages	119	±	62	107	118	±	61	114	121	±	69	116	112	±	59	116	0.386	266	±	148	257	122	±	66	117	0.234
Seasonings and spices	17	±	5	17	16	±	4	16	16	±	5	15	16	±	5	15	0.396	37	±	13	34	15	±	5	14	0.067
Women	(n=85)				(n=85)				(n=85)				(n=85)					(n=113)				(n=113)				
Cereals	119	±	24	119	124	±	24	126	121	±	25	120	122	±	23	118	0.390	214	±	50	209	122	±	27	120	0.277
Potatoes and starches	30	±	17	27	19	±	12	18	21	±	13	18	32	±	18	30	<0.001	57	±	33	50	21	±	14	19	<0.001
Sugar and sweeteners	5	±	4	4	5	±	3	4	4	±	3	4	4	±	3	3	0.079	9	±	7	7	4	±	3	4	0.095
Confectioneries	29	±	18	27	28	±	18	24	25	±	19	22	27	±	18	24	0.170	50	±	37	46	25	±	19	20	0.110
Fats and oils	5	±	2	5	4	±	2	4	5	±	2	5	4	±	2	4	0.001	9	±	5	9	6	±	3	6	0.012
Nuts and seeds	2	±	2	1	1	±	2	0	1	±	1	0	3	±	9	1	0.052	3	±	4	1	1	±	1	0	0.001
Pulses	46	±	19	42	44	±	20	39	39	±	18	36	42	±	16	40	0.016	78	±	38	75	38	±	18	35	0.026
Fish and shellfish	62	±	19	60	59	±	19	57	60	±	22	58	65	±	20	62	0.079	107	±	43	101	57	±	22	57	0.550
Meats	28	±	16	25	28	±	12	28	28	±	14	26	26	±	11	25	0.629	61	±	38	52	33	±	17	30	0.563
Eggs	18	±	8	17	19	±	9	17	19	±	11	18	19	±	9	19	0.846	32	±	15	32	19	±	10	18	0.319
Milks	76	±	49	76	89	±	53	92	95	±	57	103	74	±	55	75	<0.001	139	±	99	128	93	±	59	99	0.001
Vegetables	157	±	49	152	160	±	52	154	210	±	68	201	147	±	49	143	<0.001	288	±	106	275	195	±	70	193	<0.001
Green & yellow	60	±	28	54	61	±	29	57	82	±	47	72	42	±	23	38	<0.001	114	±	59	107	75	±	45	65	0.003
Pickled^1^	19	±	16	17	13	±	12	9	29	±	22	21	20	±	14	18	<0.001	29	±	31	17	23	±	22	17	<0.001
Fruits	85	±	45	75	73	±	43	63	101	±	65	89	110	±	51	110	<0.001	136	±	95	114	96	±	64	84	0.001
Fungi	6	±	5	5	3	±	3	2	4	±	4	3	7	±	6	5	<0.001	9	±	8	7	4	±	4	3	0.032
Algae	3	±	3	2	5	±	6	2	5	±	8	2	3	±	4	1	0.084	6	±	6	4	5	±	8	2	0.075
Alcoholic beverages	12	±	26	4	14	±	24	4	17	±	28	3	13	±	26	5	0.153	---				16	±	30	3	0.017
Non-alcoholic beverages	150	±	68	150	150	±	77	137	153	±	75	150	140	±	63	138	0.214	279	±	126	260	162	±	78	159	0.331
Seasonings and spices	19	±	5	19	20	±	6	20	19	±	6	19	19	±	5	18	0.371	33	±	12	31	18	±	6	17	0.452

^1^Pickled plum (umeboshi) was included in pickled vegetables, and not in total vegetables but rather in fruits.

^2^P-value for seasonal difference after adjusting for area difference.

Tables 9–25 showed the cumulative percent contributions of the top 20 foods for each nutrient.

**Table 9.** Cumulative % contribution of the top 20 foods for energy assessed by DR

Food code^1^	Description^1^	kcal/day	Cumulative^2^ percent
Men (n=102)			
1-41d	Rice (Paddy rice)/Grains/Well-milled rice	816.5	34.8
5-15	Fats and oils/Vegetable oils/Vegetable oil, mixed	96.9	38.9
16-2a	Fermented alcoholic beverages/Beer/Pale	83.2	42.5
16-1b	Fermented alcoholic beverages/Sake/First class	65.2	45.3
10-5a	Chicken egg/Whole egg/fresh	61.8	47.9
11-2	Liquid milk/Ordinary liquid milk	61.5	50.5
16-4b	Distilled alcoholic beverages/Shochu/25% alcohol	36.5	52.1
7-32c	Miso/Rice-koji Miso/Dark yellow type	36.2	53.6
7-21a	Tofu and Abura-age/Tofu, soybean curd/Momen	26.0	54.7
1-26a	Chinese noodles/Chinese noodle i.e., Lao Mien/Raw, dry form	23.7	55.7
1-13a	Wheat (Breads)/White bread/On the market	23.7	56.7
9-70a	Pork and separable fat/Belly without separable fat/Large-type	23.2	57.7
3-4a	Sugar/Soft sugar/White, superior	22.1	58.7
1-10a	Wheat flour/Soft flour/First grade	21.3	59.6
1-21b	Japanese noodles/Udon/Boiled	21.0	60.5
2-11a	Potatoes/Tuber/Raw	20.8	61.4
8-150a	Tunas/Bluefin tuna, raw/Lean meat	15.4	62.0
8-84a	Mackerel/Raw/Uncooked	14.6	62.6
1-24b	Japanese noodles/Somen Hiyamugi/Boiled	14.4	63.3
13-88	Apples/Raw fruit	14.3	63.9
Women (n=113)			
1-41d	Rice (Paddy rice)/Grains/Well-milled rice	523.7	28.8
5-15	Fats and oils/Vegetable oils/Vegetable oil, mixed	82.7	33.3
11-2	Liquid milk/Ordinary liquid milk	70.6	37.2
10-5a	Chicken egg/Whole egg/fresh	51.7	40.0
7-32c	Miso/Rice-koji Miso/Dark yellow type	30.9	41.7
1-13a	Wheat (Breads)/White bread/On the market	29.0	43.3
3-4a	Sugar/Soft sugar/White, superior	22.2	44.6
9-70a	Pork and separable fat/Belly without separable fat/Large-type	21.7	45.7
7-21a	Tofu and Abura-age/Tofu, soybean curd/Momen	21.3	46.9
1-10a	Wheat flour/Soft flour/First grade	19.6	48.0
2-11a	Potatoes/Tuber/Raw	19.0	49.0
1-26a	Chinese noodles/Chinese noodle i.e., Lao Mien/Raw, dry form	18.8	50.1
1-21b	Japanese noodles/Udon/Boiled	18.8	51.1
13-88	Apples/Raw fruit	17.8	52.1
17-10a	Seasonings/Mayonnaise/Whole egg type	15.7	52.9
4-24e	Japanese-style undried and semi-dried confectioneries/Manju/Mushi	13.5	53.7
4-46	Western-style undried and semi-dried confectioneries/Shortcake	12.7	54.4
7-29	Natto, fermented soybeans/Itohiki-natto	12.5	55.1
8-95a	Pacific saury/Raw/Uncooked	11.4	55.7
1-24b	Japanese noodles/Somen Hiyamugi/Boiled	11.1	56.3

^1^Food codes and descriptions correspond to those of the Standard Tables of Food Composition, 4th revised edition in Japan by Science and Technology Agency.

^2^Data on subjects in Ishikawa PHC (14-day data) were counted twice for 28-day data.

**Table 10.** Cumulative % contribution of the top 20 foods for protein assessed by DR

Food code^1^	Description^1^	g/day	Cumulative^2^ percent
Men (n=102)			
1-41d	Rice (Paddy rice)/Grains/Well-milled rice	15.6	16.8
10-5a	Chicken egg/Whole egg/fresh	4.7	21.8
8-150a	Tunas/Bluefin tuna, raw/Lean meat	3.3	25.4
11-2	Liquid milk/Ordinary liquid milk	3.0	28.6
7-32c	Miso/Rice-koji Miso/Dark yellow type	2.6	31.4
7-21a	Tofu and Abura-age/Tofu, soybean curd	2.3	33.8
8-206a	Squid and cuttlefish/Raw/Uncooked	1.8	35.8
17-3a	Seasonings/Shoyu, soy-sauce/Koikuchi	1.5	37.4
8-78a	Salmons/Mild salted chum salmon	1.4	38.9
9-47b	Chicken and separable fat/Breast	1.3	40.2
8-84a	Mackerel/Raw/Uncooked	1.2	41.6
8-95a	Pacific saury/Raw/Uncooked	1.2	42.8
7-29	Natto, fermented soybeans/Itohiki-natto	1.1	44.0
7-22	Tofu and Abura-age/Okinawa-tofu	1.1	45.2
9-68a	Pork/Loin, separable lean	1.0	46.3
8-77	Salmons/Chum salmon, raw	1.0	47.4
9-70a	Pork/Belly without separable fat	1.0	48.5
8-218	Prawns/Spiny lobster, raw	0.9	49.4
9-49b	Chicken and separable fat/Thigh/Broiler	0.9	50.4
9-72a	Pork/Inside ham, separable lean	0.9	51.4
Women (n=113)			
1-41d	Rice (Paddy rice)/Grains/Well-milled rice	10.0	13.1
10-5a	Chicken egg/Whole egg/fresh	3.9	18.3
11-2	Liquid milk/Ordinary liquid milk	3.5	22.8
7-32c	Miso/Rice-koji Miso/Dark yellow type	2.2	25.7
8-150a	Tunas/Bluefin tuna, raw/Lean meat	2.1	28.4
7-21a	Tofu and Abura-age/Tofu, soybean curd	1.9	30.9
17-3a	Seasonings/Shoyu, soy-sauce/Koikuchi	1.3	32.6
8-206a	Squid and cuttlefish/Raw/Uncooked	1.3	34.3
8-78a	Salmon/Mild salted chum salmon	1.3	35.9
7-29	Natto, fermented soybeans/Itohiki-natto	1.0	37.3
9-47b	Chicken and separable fat/Breast	1.0	38.6
8-95a	Pacific saury/Raw/Uncooked	1.0	39.9
1-13a	Wheat (Breads)/White bread/On the market	0.9	41.1
9-70a	Pork/Belly without separable fat	0.9	42.3
8-84a	Mackerel/Raw/Uncooked	0.9	43.5
8-77	Salmon/Chum salmon, raw	0.9	44.7
9-49b	Chicken and separable fat/Thigh/Broiler	0.9	45.8
7-22	Tofu and Abura-age/Okinawa-tofu	0.8	46.9
9-68a	Pork/Loin, separable lean	0.8	47.9
9-72a	Pork/Inside ham, separable lean	0.7	48.9

^1^Food codes and descriptions correspond to those of the Standard Tables of Food Composition, 4th revised edition in Japan by Science and Technology Agency.

^2^Data on subjects in Ishikawa PHC (14-day data) were counted twice for (28-day data).

**Table 11.** Cumulative % contribution of the top 20 foods for fat assessed by DR

Food code^1^	Description^1^	g/day	Cumulative^2^ percent
Men (n=102)			
5-15	Fats and oils/Vegetable oils/Vegetable oil, mixed	10.5	17.8
10-5a	Chicken egg/Whole egg/fresh	4.3	25.0
11-2	Liquid milk/Ordinary liquid milk	3.3	30.6
1-41d	Rice (Paddy rice)/Grains/Well-milled rice	3.0	35.7
9-70a	Pork and separable fat/Belly	2.0	39.1
7-21a	Tofu and Abura-age/Tofu, soybean curd	1.7	41.9
17-10a	Seasonings/Mayonnaise/Whole egg type	1.5	44.5
7-32c	Miso/Rice-koji Miso/Dark yellow type	1.1	46.3
8-84a	Mackerel/Raw/Uncooked	1.0	48.0
8-95a	Pacific saury/Raw/Uncooked	0.9	49.6
9-13b	Beef and separable fat/Frank, Plate	0.9	51.1
7-22	Tofu and Abura-age/Okinawa-tofu	0.8	52.5
9-49b	Chicken and separable fat/Thigh/Broiler	0.8	53.8
9-47b	Chicken and separable fat/Breast	0.8	55.1
7-25	Tofu and Abura-age/Abura-age	0.7	56.3
9-22	Beef and separable fat/Ground meat	0.7	57.6
9-68a	Pork and separable fat/Loin, separable lean	0.7	58.8
7-29	Natto, fermented soybeans/Itohiki-natto	0.7	59.9
11-28	Butter, salted	0.7	61.0
9-85a	Pork products/Bacon/Bacon	0.6	62.0
Women (n=113)			
5-15	Fats and oils/Vegetable oils/Vegetable oil, mixed	9.0	17.0
11-2	Liquid milk/Ordinary liquid milk	3.8	24.2
10-5a	Chicken egg/Whole egg/fresh	3.6	31.0
1-41d	Rice (Paddy rice)/Grains/Well-milled rice	1.9	34.6
9-70a	Pork and separable fat/Belly	1.9	38.1
17-10a	Seasonings/Mayonnaise/Whole egg type	1.7	41.3
7-21a	Tofu and Abura-age/Tofu, soybean curd	1.4	44.0
7-32c	Miso/Rice-koji Miso/Dark yellow type	0.9	45.7
8-95a	Pacific saury/Raw/Uncooked	0.8	47.1
8-84a	Mackerel/Raw/Uncooked	0.8	48.6
9-49b	Chicken and separable fat/Thigh/Broiler	0.7	50.0
7-25	Tofu and Abura-age/Abura-age	0.7	51.3
11-28	Butter, salted	0.7	52.6
7-22	Tofu and Abura-age/Okinawa-tofu	0.7	53.9
9-13b	Beef and separable fat/Frank, Short plate	0.6	55.0
7-29	Natto, fermented soybeans/Itohiki-natto	0.6	56.2
9-47b	Chicken and separable fat/Breast	0.6	57.3
9-87f	Pork products/Sausage/Lyoner	0.6	58.4
9-85a	Pork products/Bacon/Bacon	0.6	59.5
5-7a	Margarine/Soft type	0.6	60.5

^1^Food codes and descriptions correspond to those of the Standard Tables of Food Composition, 4th revised edition in Japan by Science and Technology Agency.

^2^Data on subjects in Ishikawa PHC (14-day data) were counted twice for (28-day data).

**Table 12.** Cumulative % contribution of the top 20 foods for carbohydrate assessed by DR

Food code^1^	Description^1^	g/day	Cumulative^2^ percent
Men (n=102)			
1-41d	Rice (Paddy rice)/Grains/Well-milled rice	173.8	54.9
16-2a	Beer/Pale	6.6	56.9
3-4a	Sugar/Soft sugar/White, superior	5.7	58.7
11-2	Liquid milk/Ordinary liquid milk	4.7	60.2
1-26a	Chinese noodle i.e., Lao Mien/Raw, dry form	4.7	61.7
2-11a	Potatoes/Tuber/Raw	4.6	63.2
1-10a	Wheat flour/Soft flour/First grade	4.4	64.6
1-13a	Wheat (Breads)/White bread/On the market	4.4	65.9
1-21b	Japanese noodles/Udon/Boiled	4.3	67.3
7-32c	Miso/Rice-koji Miso/Dark yellow type	4.1	68.6
13-88	Apples/Raw fruit	3.9	69.8
16-1b	Sake/First class	3.0	70.7
1-24b	Japanese noodles/Somen Hiyamugi/Boiled	2.9	71.7
13-64	Bananas/Raw fruit	2.2	72.3
16-31	Other beverages/Coffee drink, canned	2.1	73.0
1-62a	Dried buckwheat noodle/Raw, dry form	2.0	73.6
1-47	Mochi, glutinous rice cake	1.8	74.2
12-18a	Pumpkin and squash/Squash/Raw	1.6	74.7
4-24e	Japanese-style confectioneries/Manju	1.6	75.2
13-26a	Japanese Persimmons/Raw fruit/Hard type	1.6	75.7
Women (n=113)			
1-41d	Rice (Paddy rice)/Grains/Well-milled rice	111.5	43.4
3-4a	Sugar/Soft sugar/White, superior	5.7	45.6
11-2	Liquid milk/Ordinary liquid milk	5.4	47.7
1-13a	Wheat (Breads)/White bread/On the market	5.4	49.8
13-88	Apples/Raw fruit	4.8	51.7
2-11a	Potatoes/Tuber/Raw	4.2	53.3
1-10a	Wheat flour/Soft flour/First grade	4.1	54.9
1-21b	Japanese noodles/Udon/Boiled	3.8	56.4
1-26a	Chinese noodle i.e., Lao Mien/Raw, dry form	3.7	57.8
7-32c	Miso/Rice-koji Miso/Dark yellow type	3.5	59.2
4-24e	Japanese-style confectioneries/Manju	3.1	60.4
13-64	Bananas/Raw fruit	2.3	61.3
1-24b	Japanese noodles/Somen Hiyamugi/Boiled	2.2	62.1
12-18a	Pumpkin and squash/Squash/Raw	2.1	62.9
2-5a	Sweet potatoes/Tuber/Raw	2.0	63.7
13-26a	Japanese Persimmons/Raw fruit/Hard type	2.0	64.5
13-17b	Satsuma mandarins/Raw fruit	1.9	65.3
4-46	Confectioneries/Shortcake	1.8	66.0
4-41d	Confectioneries/Rice-cracker	1.7	66.6
4-54	Japanese buns/An-pan, bean-jam bun	1.7	67.3

^1^Food codes and descriptions correspond to those of the Standard Tables of Food Composition, 4th revised edition in Japan by Science and Technology Agency.

^2^Data on subjects in Ishikawa PHC (14-day data) were counted twice for (28-day data).

**Table 13.** Cumulative % contribution of the top 20 foods for alcohol assessed by DR

Food code^1^	Description^1^	g/day	Cumulative^2^ percent
Men (n=102)			
16-2a	Beer/Pale	7.7	34.0
16-1b	Sake/First class	7.5	67.4
16-4b	Shochu/25% alcohol	5.3	90.8
16-5a	Whisky/Special class	0.6	93.5
16-5b	Whisky/First class	0.6	96.0
16-1c	Sake/Second class	0.3	97.5
16-17a	Mirin/Hon-mirin	0.2	98.6
16-4c	Shochu/20% alcohol	0.1	99.0
16-3a	Wine/White	0.1	99.2
16-10	Umeshu	0.0	99.4
16-6b	Brandy/First class	0.0	99.6
17-33a	Sakekasu/Seisyu	0.0	99.7
16-.	Sake/Special class	0.0	99.8
16-3b	Wine/Red	0.0	99.9
16-12	Sweet wine	0.0	99.9
16-4a	Shochu/35% alcohol	0.0	99.9
16-18	Medical liqueur	0.0	100.0
16-16	Shiro-zake	0.0	100.0
16-6c	Brandy/Second class	0.0	100.0
---	---		
Women (n=113)			
16-2a	Beer/Pale	0.5	33.5
16-1b	Sake/First class	0.4	56.3
16-1c	Sake/Second class	0.3	72.8
16-17a	Mirin/Hon-mirin	0.2	85.8
16-10	Umeshu	0.0	88.4
17-33a	Sakekasu/Seisyu	0.0	90.3
16-3a	Wine/White	0.0	92.1
16-4c	Shochu/20% alcohol	0.0	94.0
16-5a	Whisky/Special class	0.0	95.2
16-18	Medical liqueur	0.0	96.4
16-6a	Brandy/Special class	0.0	97.3
16-4b	Shochu/25% alcohol	0.0	98.2
16-4a	Shochu/35% alcohol	0.0	98.8
16-3b	Wine/Red	0.0	99.4
16-12	Sweet wine	0.0	99.9
16-6b	Brandy/First class	0.0	99.9
16-9	Rum	0.0	100.0
16-14a	Curacao/Orange	0.0	100.0
16-6c	Brandy/Second class	0.0	100.0
^---^	^---^		

^1^Food codes and descriptions correspond to those of the Standard Tables of Food Composition, 4th revised edition in Japan by Science and Technology Agency.

^2^Data on subjects in Ishikawa PHC (14-day data) were counted twice for (28-day data).

**Table 14.** Cumulative % contribution of the top 20 foods for calcium assessed by DR

Food code^1^	Description^1^	mg/day	Cumulative^2^ percent
Men (n=102)			
11-2	Liquid milk/Ordinary liquid milk	104.2	16.7
7-21a	Tofu and Abura-age/Tofu, soybean curd	40.5	23.2
7-32c	Miso/Rice-koji Miso/Dark yellow type	25.3	27.3
10-5a	Chicken egg/Whole egg/fresh	21.0	30.7
8-31	Sardines/Boiled and dried	15.2	33.1
7-22	Tofu and Abura-age/Okinawa-tofu	14.1	35.3
1-41d	Rice (Paddy rice)/Grains/Well-milled rice	13.8	37.6
12-24a	Cabbage/Head/Raw	11.6	39.4
11-23	Cheeses/Process cheeses	10.5	41.1
12-56a	Daikon, Japanese radish/Root/Raw	9.7	42.7
12-98b	Nozawana/Leaves/Salted	8.4	44.0
12-94a	Carrot/Root/Raw	8.2	45.3
12-32a	Komatsuna/Leaves/Raw	8.1	46.6
15-35a	Wakame/Dried/Dried	7.9	47.9
7-25	Tofu and Abura-age/Abura-age	6.7	49.0
7-27	Tofu and Abura-age/Kori-dofu	6.4	50.0
12-117a	Spinach/Leaves/Raw	6.3	51.0
7-29	Natto, fermented soybeans/Itohiki-natto	6.2	52.0
11-9a	Yogurt/Whole milk, unsweetened	5.5	52.9
16-31	Other beverages/Coffee drink, canned	5.5	53.8
Women (n=113)			
11-2	Liquid milk/Ordinary liquid milk	119.7	20.0
7-21a	Tofu and Abura-age/Tofu, soybean curd	33.2	25.5
7-32c	Miso/Rice-koji Miso/Dark yellow type	21.6	29.1
10-5a	Chicken egg/Whole egg/fresh	17.5	32.0
8-31	Sardines/Boiled and dried	13.4	34.3
7-22	Tofu and Abura-age/Okinawa-tofu	11.0	36.1
12-24a	Cabbage/Head/Raw	10.2	37.8
11-23	Cheeses/Process cheeses	9.8	39.4
1-41d	Rice (Paddy rice)/Grains/Well-milled rice	8.8	40.9
11-9b	Yogurt/Partially skimmed, sweetened	8.8	42.4
12-56a	Daikon, Japanese radish/Root/Raw	8.6	43.8
15-35a	Wakame/Dried/Dried	8.4	45.2
12-32a	Komatsuna/Leaves/Raw	8.1	46.6
12-94a	Carrot/Root/Raw	7.6	47.8
12-98b	Nozawana/Leaves/Salted	7.0	49.0
7-25	Tofu and Abura-age/Abura-age	6.5	50.1
6-12b	Nuts and Seeds/Sesame seeds/Roasted	6.5	51.2
12-117a	Spinach/Leaves/Raw	6.4	52.2
11-9a	Yogurt/Whole milk, unsweetened	5.9	53.2
7-29	Natto, fermented soybeans/Itohiki-natto	5.6	54.2

^1^Food codes and descriptions correspond to those of the Standard Tables of Food Composition, 4th revised edition in Japan by Science and Technology Agency.

^2^Data on subjects in Ishikawa PHC (14-day data) were counted twice for (28-day data).

**Table 15.** Cumulative % contribution of the top 20 foods for phosphorus assessed by DR

Food code^1^	Description^1^	mg/day	Cumulative^2^ percent
Men (n=102)			
1-41d	Rice (Paddy rice)/Grains/Well-milled rice	321.1	22.7
11-2	Liquid milk/Ordinary liquid milk	93.8	29.3
10-5a	Chicken egg/Whole egg/fresh	76.3	34.7
7-32c	Miso/Rice-koji Miso/Dark yellow type	39.0	37.5
8-150a	Tuna/Bluefin tuna, raw/Lean meat	32.5	39.8
16-2a	Beer/Pale	29.9	41.9
7-21a	Tofu and Abura-age/Tofu, soybean curd	28.7	43.9
17-3a	Seasonings/Shoyu, soy-sauce/Koikuchi	28.3	45.9
8-206a	Squid and cuttlefish/Raw/Uncooked	19.4	47.3
7-22	Tofu and Abura-age/Okinawa-tofu	15.2	48.4
2-11a	Potatoes/Tuber/Raw	14.8	49.4
8-78a	Mild salted chum salmon/Uncooked	14.0	50.4
7-29	Natto, fermented soybeans/Itohiki-natto	13.1	51.3
11-23	Cheeses/Process cheeses	12.2	52.2
8-218	Spiny lobster, raw	11.7	53.0
9-47b	Chicken and separable fat/Breast	11.0	53.8
8-31	Sardines/Boiled and dried	10.4	54.5
8-77	Salmon/Chum salmon, raw	10.2	55.3
8-84a	Mackerel/Raw/Uncooked	9.8	56.0
8-95a	Pacific saury/Raw/Uncooked	9.1	56.6
Women (n=113)			
1-41d	Rice (Paddy rice)/Grains/Well-milled rice	205.9	17.6
11-2	Liquid milk/Ordinary liquid milk	107.7	26.8
10-5a	Chicken egg/Whole egg/fresh	63.8	32.2
7-32c	Miso/Rice-koji Miso/Dark yellow type	33.3	35.0
17-3a	Seasonings/Shoyu, soy-sauce/Koikuchi	24.6	37.1
7-21a	Tofu and Abura-age/Tofu, soybean curd	23.5	39.1
8-150a	Tuna/Bluefin tuna, raw/Lean meat	20.5	40.9
8-206a	Squid and cuttlefish/Raw/Uncooked	13.8	42.1
2-11a	Potatoes/Tuber/Raw	13.6	43.2
8-78a	Mild salted chum salmon/Uncooked	12.7	44.3
7-22	Tofu and Abura-age/Okinawa-tofu	11.9	45.3
7-29	Natto, fermented soybeans/Itohiki-natto	11.9	46.3
11-23	Cheeses/Process cheeses	11.4	47.3
8-31	Sardines/Boiled and dried	9.2	48.1
8-218	Spiny lobster, raw	9.0	48.9
8-77	Salmon/Chum salmon,raw	8.9	49.6
9-47b	Chicken and separable fat/Breast	8.6	50.4
1-13a	Wheat (Breads)/White bread/On the market	7.8	51.0
8-95a	Pacific saury/Raw/Uncooked	7.6	51.7
11-9b	Yogurt/Partially skimmed, sweetened	7.5	52.3

^1^Food codes and descriptions correspond to those of the Standard Tables of Food Composition, 4th revised edition in Japan by Science and Technology Agency.

^2^Data on subjects in Ishikawa PHC (14-day data) were counted twice for (28-day data).

**Table 16.** Cumulative % contribution of the top 20 foods for iron assessed by DR

Food code^1^	Description^1^	mg/day	Cumulative^2^ percent
Men (n=102)			
1-41d	Rice (Paddy rice)/Grains/Well-milled rice	1.1	8.9
7-32c	Miso/Rice-koji Miso/Dark yellow type	0.8	15.4
10-5a	Chicken egg/Whole egg/fresh	0.7	20.8
7-21a	Tofu and Abura-age/Tofu, soybean curd	0.5	24.5
17-3a	Seasonings/Shoyu,soy-sauce/Koikuchi	0.5	28.1
12-117a	Spinach/Leaves/Raw	0.4	31.4
8-150a	Tuna/Bluefin tuna, raw/Lean meat	0.2	33.2
7-29	Natto, fermented soybeans/Itohiki-natto	0.2	34.9
7-22	Tofu and Abura-age/Okinawa-tofu	0.2	36.5
12-94a	Carrot/Root/Raw	0.2	37.8
9-80	Swine(Sweetbreads)/Liver	0.2	39.1
15-28	Hijiki/Boiled and dried	0.2	40.4
16-21b	Green tea/Sencha, common grade/Infusion	0.1	41.5
2-11a	Potatoes/Tuber/Raw	0.1	42.6
8-31	Sardines/Boiled and dried	0.1	43.5
12-24a	Cabbage/Head/Raw	0.1	44.4
11-2	Liquid milk/Ordinary liquid milk	0.1	45.2
7-27	Tofu and Abura-age/Kori-dofu	0.1	46.0
12-56a	Daikon, Japanese radish/Root/Raw	0.1	46.7
7-25	Tofu and Abura-age/Abura-age	0.1	47.5
Women (n=113)			
1-41d	Rice (Paddy rice)/Grains/Well-milled rice	0.7	6.5
7-32c	Miso/Rice-koji Miso/Dark yellow type	0.7	12.9
10-5a	Chicken egg/Whole egg/fresh	0.6	18.0
12-117a	Spinach/Leaves/Raw	0.4	21.8
17-3a	Seasonings/Shoyu,soy-sauce/Koikuchi	0.4	25.4
7-21a	Tofu and Abura-age/Tofu, soybean curd	0.4	28.8
7-29	Natto, fermented soybeans/Itohiki-natto	0.2	30.7
7-22	Tofu and Abura-age/Okinawa-tofu	0.2	32.0
12-94a	Carrot/Root/Raw	0.2	33.4
15-28	Hijiki/Boiled and dried	0.2	34.8
16-21b	Green tea/Sencha, common grade/Infusion	0.1	36.1
8-150a	Tuna/Bluefin tuna, raw/Lean meat	0.1	37.4
2-11a	Potatoes/Tuber/Raw	0.1	38.5
11-2	Liquid milk/Ordinary liquid milk	0.1	39.5
1-13a	Wheat (Breads)/White bread/On the market	0.1	40.5
8-31	Sardines/Boiled and dried	0.1	41.5
12-24a	Cabbage/Head/Raw	0.1	42.4
7-25	Tofu and Abura-age/Abura-age	0.1	43.2
7-27	Tofu and Abura-age/Kori-dofu	0.1	43.9
9-80	Swine(Sweetbreads)/Liver	0.1	44.7

^1^Food codes and descriptions correspond to those of the Standard Tables of Food Composition, 4th revised edition in Japan by Science and Technology Agency.

^2^Data on subjects in Ishikawa PHC (14-day data) were counted twice for (28-day data).

**Table 17.** Cumulative % contribution of the top 20 foods for sodium assessed by DR

Food code^1^	Description^1^	mg/day	Cumulative^2^ percent
Men (n=102)			
17-3a	Seasonings/Shoyu, soy-sauce/Koikuchi	1195	22.4
7-32c	Miso/Rice-koji Miso/Dark yellow type	994	41.0
17-2a	Seasonings/Salts/Kitchen salt	542	51.2
17-8	Seasonings/Flavor seasoning	171	54.4
12-58	Daikon, Japanese radish/Takuan-zuke	146	57.1
8-78a	Mild salted chum salmon/Uncooked	140	59.8
13-14	Ume/Umeboshi, salted and dried	100	61.6
13-13	Ume/Umezuke, salted	78	63.1
17-11	Seasonings/Mentsuyu	78	64.6
12-25b	Cucumber/Fruit/Salted	75	66.0
1-31b	Precooked Chinese noodles/Dried by frying	62	67.1
17-16b	Spices/Curry/Roux	54	68.2
8-81	Salmon/Salted roe	54	69.2
11-2	Liquid milk/Ordinary liquid milk	52	70.1
15-35a	Wakame/Dried/Dried	50	71.1
10-5a	Chicken egg/Whole egg/fresh	50	72.0
1-13a	Wheat (Breads)/White bread/On the market	47	72.9
12-98b	Nozawana/Leaves/Salted	43	73.7
8-250	Fish paste products/Yaki-chikuwa, broiled	40	74.5
8-212	Squid and cuttlefish/Salted preserves	36	75.1
Women (n=113)			
17-3a	Seasonings/Shoyu, soy-sauce/Koikuchi	1038	22.3
7-32c	Miso/Rice-koji Miso/Dark yellow type	848	40.5
17-2a	Seasonings/Salts/Kitchen salt	478	50.8
17-8	Seasonings/Flavor seasoning	141	53.8
8-78a	Mild salted chum salmon/Uncooked	127	56.6
12-58	Daikon, Japanese radish/Takuan-zuke	123	59.2
13-14	Ume/Umeboshi, salted and dried	77	60.9
17-11	Seasonings/Mentsuyu	74	62.5
12-25b	Cucumber/Fruit/Salted	73	64.0
13-13	Ume/Umezuke, salted	62	65.4
11-2	Liquid milk/Ordinary liquid milk	60	66.6
1-13a	Wheat (Breads)/White bread/On the market	58	67.9
15-35a	Wakame/Dried/Dried	53	69.0
17-16b	Spices/Curry/Roux	49	70.1
8-81	Salmon/Salted roe	45	71.1
10-5a	Chicken egg/Whole egg/fresh	41	72.0
1-31b	Precooked Chinese noodles/Dried by frying	40	72.8
17-1	Seasonings/Consomme, dried	39	73.7
8-250	Fish paste products/Yaki-chikuwa, broiled	39	74.5
12-98b	Nozawana/Leaves/Salted	36	75.3

^1^Food codes and descriptions correspond to those of the Standard Tables of Food Composition, 4th revised edition in Japan by Science and Technology Agency.

^2^Data on subjects in Ishikawa PHC (14-day data) were counted twice for (28-day data).

**Table 18.** Cumulative % contribution of the top 20 foods for potassium assessed by DR

Food code^1^	Description^1^	mg/day	Cumulative^2^ percent
Men (n=102)			
1-41d	Rice (Paddy rice)/Grains/Well-milled rice	252	7.8
11-2	Liquid milk/Ordinary liquid milk	156	12.7
2-11a	Potatoes/Tuber/Raw	121	16.5
7-32c	Miso/Rice-koji Miso/Dark yellow type	86	19.1
12-117a	Spinach/Leaves/Raw	85	21.8
12-94a	Carrot/Root/Raw	84	24.4
17-3a	Seasonings/Shoyu, soy-sauce/Koikuchi	81	26.9
12-56a	Daikon, Japanese radish/Root/Raw	78	29.3
16-2a	Fermented alcoholic beverages/Beer/Pale	75	31.7
12-24a	Cabbage/Head/Raw	57	33.4
8-150a	Tunas/Bluefin tuna, raw/Lean meat	49	34.9
10-5a	Chicken egg/Whole egg/fresh	46	36.4
15-35a	Wakame/Dried/Dried	45	37.8
7-29	Natto, fermented soybeans/Itohiki-natto	45	39.2
12-85	Tomatoes/Fruit	40	40.4
13-64	Bananas/Raw fruit	37	41.6
15-17	Konbu, kelp/Rishiri-konbu, dried	36	42.7
8-206a	Squid and cuttlefish/Raw/Uncooked	33	43.7
12-18a	Pumpkin and squash/Squash/Raw	32	44.7
12-25a	Cucumber/Fruit/Raw	32	45.7
Women (n=113)			
11-2	Liquid milk/Ordinary liquid milk	179	6.1
1-41d	Rice (Paddy rice)/Grains/Well-milled rice	162	11.6
2-11a	Potatoes/Tuber/Raw	111	15.3
12-117a	Spinach/Leaves/Raw	86	18.3
12-94a	Carrot/Root/Raw	78	20.9
7-32c	Miso/Rice-koji Miso/Dark yellow type	73	23.4
17-3a	Seasonings/Shoyu, soy-sauce/Koikuchi	70	25.8
12-56a	Daikon, Japanese radish/Root/Raw	69	28.1
12-85	Tomatoes/Fruit	55	30.0
12-24a	Cabbage/Head/Raw	50	31.6
15-35a	Wakame/Dried/Dried	48	33.3
7-29	Natto, fermented soybeans/Itohiki-natto	41	34.7
12-18a	Pumpkin and squash/Squash/Raw	41	36.1
13-88	Apples/Raw fruit	39	37.4
13-64	Bananas/Raw fruit	39	38.7
10-5a	Chicken egg/Whole egg/fresh	38	40.0
15-17	Konbu, kelp/Rishiri-konbu, dried	33	41.1
2-5a	Sweet potatoes/Tuber/Raw	32	42.2
12-25a	Cucumber/Fruit/Raw	32	43.3
8-150a	Tuna/Bluefin tuna, raw/Lean meat	31	44.3

^1^Food codes and descriptions correspond to those of the Standard Tables of Food Composition, 4th revised edition in Japan by Science and Technology Agency.

^2^Data on subjects in Ishikawa PHC (14-day data) were counted twice for (28-day data).

**Table 19.** Cumulative % contribution of the top 20 foods for retinol assessed by DR

Food code^1^	Description^1^	*µ*g/day	Cumulative^2^ percent
Men (n=102)			
9-80	Swine (Sweetbreads)/Liver	168.6	38.4
10-5a	Chicken egg/Whole egg/fresh	72.5	54.9
9-55	Chicken (Sweetbreads)/Liver	67.4	70.2
11-2	Liquid milk/Ordinary liquid milk	28.1	76.7
8-69	Sablefish/Raw	20.6	81.4
8-41	Eel/Kabayaki	17.5	85.3
11-28	Butter, salted	4.0	86.3
11-23	Cheeses/Process cheeses	4.0	87.2
8-8a	Conger myriaster (Brevoort)	3.5	88.0
8-77	Salmon/Chum salmon, raw	2.9	88.6
9-47b	Chicken and separable fat/Breast	2.5	89.2
8-16	Angler fish/Liver	2.3	89.7
9-49b	Chicken and separable fat/Thigh/Broiler	2.1	90.2
8-81	Salmon/Salted roe	2.1	90.7
8-95a	Pacific saury/Raw/Uncooked	2.1	91.2
8-84a	Mackerel/Raw/Uncooked	1.8	91.6
4-46	Confectioneries/Shortcake	1.8	92.0
8-124	Herring/Raw	1.6	92.3
8-241a	Toyama squid/Raw/Uncooked	1.5	92.7
9-54	Chicken (Sweetbreads)/Heart	1.5	93.0
Women (n=113)			
9-55	Chicken (Sweetbreads)/Liver	96.6	26.1
9-80	Swine (Sweetbreads)/Liver	88.0	50.0
10-5a	Chicken egg/Whole egg/fresh	60.6	66.4
11-2	Liquid milk/Ordinary liquid milk	32.3	75.1
8-69	Sablefish/Raw	14.2	78.9
8-41	Eel/Kabayaki	12.3	82.3
9-26	Cattle (Sweetbreads)/Liver	5.7	83.8
11-28	Butter, salted	4.2	84.9
11-23	Cheeses/Process cheeses	3.7	85.9
4-46	Confectioneries/Shortcake	3.5	86.9
8-8a	Conger myriaster (Brevoort)	2.8	87.7
8-77	Salmon/Chum salmon, raw	2.5	88.4
9-49b	Chicken and separable fat/Thigh/Broiler	2.0	88.9
4-7	Japanese-style confectioneries/Kasutera	1.9	89.4
9-47b	Chicken and separable fat/Breast	1.9	89.9
8-81	Salmon/Salted roe	1.8	90.4
8-95a	Pacific saury/Raw/Uncooked	1.7	90.9
9-54	Chicken (Sweetbreads)/Heart	1.5	91.3
8-84a	Mackerel/Raw/Uncooked	1.4	91.7
11-9a	Yogurt/Whole milk, unsweetened	1.3	92.0

^1^Food codes and descriptions correspond to those of the Standard Tables of Food Composition, 4th revised edition in Japan by Science and Technology Agency.

^2^Data on subjects in Ishikawa PHC (14-day data) were counted twice for (28-day data).

**Table 20.** Cumulative % contribution of the top 20 foods for carotene assessed by DR

Food code^1^	Description^1^	*µ*g/day	Cumulative^2^ percent
Men (n=102)			
12-94a	Carrot/Root/Raw	1539	46.9
12-117a	Spinach/Leaves/Raw	355	57.7
12-93a	Chinese chive/Leaves/Raw	112	61.2
12-98b	Nozawana/Leaves/Salted	103	64.3
12-32a	Komatsuna/Leaves/Raw	93	67.1
12-18a	Pumpkin and squash/Squash/Raw	74	69.4
12-85	Tomatoes/Fruit	68	71.5
15-4	Purple laver/Toasted	60	73.3
12-39a	Garland chrysanthemum/Leaves/Raw	57	75.1
12-55a	Daikon, Japanese radish/Leaves/Raw	44	76.4
12-19a	Leaf mustard/stems and leaves/Raw	39	77.6
13-79	Mangos/Raw fruit	37	78.7
15-5	Purple laver/Seasoned and dried	35	79.8
12-77a	Basella/Leaves/Raw	33	80.8
12-74a	Chingentsuai/Leaves/Raw	27	81.6
15-35a	Wakame/Dried/Dried	27	82.5
12-72	Lettuce/Head lettuce, butter head type	26	83.2
12-114a	Broccoli/Head/Raw	26	84.0
13-45	Watermelon/Raw fruit	24	84.8
12-76	New Zealand spinach/stems and leaves	23	85.5
Women (n=113)			
12-94a	Carrot/Root/Raw	1422	44.6
12-117a	Spinach/Leaves/Raw	361	55.9
12-93a	Chinese chive/Leaves/Raw	103	59.2
12-18a	Pumpkin and squash/Squash/Raw	93	62.1
12-85	Tomatoes/Fruit	93	65.0
12-32a	Komatsuna/Leaves/Raw	92	67.9
12-98b	Nozawana/Leaves/Salted	87	70.6
15-4	Purple laver/Toasted	67	72.7
12-39a	Garland chrysanthemum/Leaves/Raw	54	74.4
13-79	Mangos/Raw fruit	47	75.9
12-77a	Basella/Leaves/Raw	43	77.3
12-55a	Daikon, Japanese radish/Leaves/Raw	43	78.6
15-5	Purple laver/Seasoned and dried	31	79.6
13-45	Watermelon/Raw fruit	29	80.5
15-35a	Wakame/Dried/Dried	29	81.4
12-114a	Broccoli/Head/Raw	29	82.3
12-74a	Chingentsuai/Leaves/Raw	27	83.1
12-19a	Leaf mustard/stems and leaves/Raw	25	83.9
12-113a	Chard, Swiss chard/Leaves/Raw	24	84.7
12-25a	Cucumber/Fruit/Raw	23	85.4

^1^Food codes and descriptions correspond to those of the Standard Tables of Food Composition, 4th revised edition in Japan by Science and Technology Agency.

^2^Data on subjects in Ishikawa PHC (14-day data) were counted twice for (28-day data).

**Table 21.** Cumulative % contribution of the top 20 foods for thiamin assessed by DR

Food code^1^	Description^1^	mg/day	Cumulative^2^ percent
Men (n=102)			
1-41d	Rice (Paddy rice)/Grains/Well-milled rice	0.28	20.9
9-68a	Pork and separable fat/Loin, separable lean	0.05	25.0
9-72a	Pork/Inside ham, separable lean	0.05	28.9
9-70a	Pork/Belly without separable fat	0.05	32.4
11-2	Liquid milk/Ordinary liquid milk	0.03	34.8
10-5a	Chicken egg/Whole egg/fresh	0.03	37.1
2-11a	Potatoes/Tuber/Raw	0.03	39.3
1-41f	Enriched rice with embryo	0.02	41.2
7-21a	Tofu and Abura-age/Tofu, soybean curd	0.02	43.0
9-66a	Pork/Boston butt, separable lean	0.02	44.7
9-86c	Pork products/Ham/Loin	0.02	46.1
9-75a	Pork and separable fat/Fillet	0.02	47.5
9-76	Pork and separable fat/Ground meat	0.02	48.6
12-117a	Spinach/Leaves/Raw	0.01	49.7
12-94a	Carrot/Root/Raw	0.01	50.9
9-84	Pork products/Roasted pork	0.01	51.9
12-24a	Cabbage/Head/Raw	0.01	52.9
13-17b	Satsuma mandarins	0.01	53.8
7-22	Tofu and Abura-age/Okinawa-tofu	0.01	54.7
8-150a	Tuna/Bluefin tuna, raw/Lean meat	0.01	55.6
Women (n=113)			
1-41d	Rice (Paddy rice)/Grains/Well-milled rice	0.15	15.7
9-70a	Pork/Belly without separable fat	0.04	19.6
9-68a	Pork and separable fat/Loin, separable lean	0.04	23.3
9-72a	Pork/Inside ham, separable lean	0.04	26.9
11-2	Liquid milk/Ordinary liquid milk	0.03	30.1
2-11a	Potatoes/Tuber/Raw	0.02	32.5
10-5a	Chicken egg/Whole egg/fresh	0.02	34.8
9-66a	Pork/Boston butt, separable lean	0.02	36.6
7-21a	Tofu and Abura-age/Tofu, soybean curd	0.02	38.3
1-41f	Enriched rice with embryo	0.02	40.0
13-17b	Satsuma mandarins	0.01	41.5
9-86c	Pork products/Ham/Loin	0.01	43.0
9-75a	Pork and separable fat/Fillet	0.01	44.4
12-117a	Spinach/Leaves/Raw	0.01	45.7
9-76	Pork and separable fat/Ground meat	0.01	46.9
12-94a	Carrot/Root/Raw	0.01	48.2
12-24a	Cabbage/Head/Raw	0.01	49.2
12-85	Tomatoes/Fruit	0.01	50.3
12-18a	Pumpkin and squash/Squash/Raw	0.01	51.3
8-78a	Mild salted chum salmon/Uncooked	0.01	52.1

^1^Food codes and descriptions correspond to those of the Standard Tables of Food Composition, 4th revised edition in Japan by Science and Technology Agency.

^2^Data on subjects in Ishikawa PHC (14-day data) were counted twice for (28-day data).

**Table 22.** Cumulative % contribution of the top 20 foods for riboflavin assessed by DR

Food code^1^	Description^1^	mg/day	Cumulative^2^ percent
Men (n=102)			
10-5a	Chicken egg/Whole egg/fresh	0.18	11.8
11-2	Liquid milk/Ordinary liquid milk	0.16	21.8
1-41d	Rice (Paddy rice)/Grains/Well-milled rice	0.07	26.3
16-2a	Beer/Pale	0.06	30.4
9-80	Swine (Sweetbreads)/Liver	0.05	33.4
16-21b	Green tea/Sencha, common grade/Infusion	0.04	36.2
7-29	Natto, fermented soybeans/Itohiki-natto	0.04	38.7
17-3a	Seasonings/Shoyu, soy-sauce/Koikuchi	0.04	41.2
8-84a	Mackerel/Raw/Uncooked	0.03	43.3
12-117a	Spinach/Leaves/Raw	0.03	45.0
7-32c	Miso/Rice-koji Miso/Dark yellow type	0.02	46.2
8-95a	Pacific saury/Raw/Uncooked	0.02	47.5
14-7a	Shiitake/Dried/Uncooked	0.02	48.4
12-24a	Cabbage/Head/Raw	0.01	49.3
9-70a	Pork/Belly without separable fat	0.01	50.1
8-60a	Flatfish/Raw/Uncooked	0.01	50.9
8-78a	Mild salted chum salmon/Uncooked	0.01	51.7
9-49b	Chicken and separable fat/Thigh/Broiler	0.01	52.4
9-72a	Pork/Inside ham, separable lean	0.01	53.1
16-31	Other beverages/Coffee drink, canned	0.01	53.8
Women (n=113)			
11-2	Liquid milk/Ordinary liquid milk	0.18	13.0
10-5a	Chicken egg/Whole egg/fresh	0.15	24.1
16-21b	Green tea/Sencha, common grade/Infusion	0.04	27.3
1-41d	Rice (Paddy rice)/Grains/Well-milled rice	0.04	30.5
7-29	Natto, fermented soybeans/Itohiki-natto	0.04	33.0
17-3a	Seasonings/Shoyu, soy-sauce/Koikuchi	0.03	35.5
12-117a	Spinach/Leaves/Raw	0.03	37.4
8-84a	Mackerel/Raw/Uncooked	0.02	39.2
9-80	Swine (Sweetbreads)/Liver	0.02	41.0
7-32c	Miso/Rice-koji Miso/Dark yellow type	0.02	42.2
8-95a	Pacific saury/Raw/Uncooked	0.02	43.3
14-7a	Shiitake/Dned/Uncooked	0.01	44.4
11-9b	Yogurt/Partially skimmed, sweetened	0.01	45.3
9-55	Chicken (Sweetbreads)/Liver	0.01	46.2
16-26b	Fermented tea/Oolong tea/Infusion	0.01	47.1
12-24a	Cabbage/Head/Raw	0.01	48.0
9-70a	Pork/Belly without separable fat	0.01	48.8
9-49b	Chicken and separable fat/Thigh/Broiler	0.01	49.6
8-78a	Mild salted chum salmon/Uncooked	0.01	50.4
12-114a	Broccoli/Head/Raw	0.01	51.2

^1^Food codes and descriptions correspond to those of the Standard Tables of Food Composition, 4th revised edition in Japan by Science and Technology Agency.

^2^Data on subjects in Ishikawa PHC (14-day data) were counted twice for (28-day data).

**Table 23.** Cumulative % contribution of the top 20 foods for niacin assessed by DR

Food code^1^	Description^1^	mg/day	Cumulative^2^ percent
Men (n=102)			
1-41d	Rice (Paddy rice)/Grains/Well-milled rice	3.2	14.7
16-2a	Beer/Pale	1.3	20.5
8-150a	Tuna/Bluefin tuna, raw/Lean meat	1.2	25.8
8-84a	Mackerel/Raw/Uncooked	0.6	28.6
9-47b	Chicken and separable fat/Breast	0.6	31.3
8-50	Skipjack and frigate mackerel/Skipjack, raw	0.6	33.8
2-11a	Potatoes/Tuber/Raw	0.5	36.0
8-77	Salmon/Chum salmon, raw	0.4	37.9
8-78a	Mild salted chum salmon/Uncooked	0.4	39.6
9-68a	Pork and separable fat/Loin, separable lean	0.4	41.2
9-70a	Pork/Belly without separable fat	0.3	42.8
9-72a	Pork/Inside ham, separable lean	0.3	44.4
8-206a	Squid and cuttlefish/Raw/Uncooked	0.3	45.9
8-54	Skipjack/Katsuo-bushi, dried strip	0.3	47.3
8-153c	Tuna/Canned with/Oil, solids and liquid	0.3	48.8
8-95a	Pacific saury/Raw/Uncooked	0.3	50.1
7-32c	Miso/Rice-koji Miso/Dark yellow type	0.3	51.5
14-10a	Shimeji/Shirotamogitake, raw/Uncooked	0.2	52.5
17-3a	Seasonings/Shoyu, soy-sauce/Koikuchi	0.2	53.5
9-84	Pork products/Roasted pork	0.2	54.5
Women (n=113)			
1-41d	Rice (Paddy rice)/Grains/Well-milled rice	2.1	12.2
8-150a	Tuna/Bluefin tuna, raw/Lean meat	0.7	16.5
9-47b	Chicken and separable fat/Breast	0.5	19.2
8-84a	Mackerel/Raw/Uncooked	0.4	21.8
2-11a	Potatoes/Tuber/Raw	0.4	24.5
8-50	Skipjack and frigate mackerel/Skipjack, raw	0.4	26.7
8-77	Salmon/Chum salmon, raw	0.4	28.8
8-78a	Mild salted chum salmon/Uncooked	0.3	30.8
9-70a	Pork/Belly without separable fat	0.3	32.7
9-72a	Pork/Inside ham, separable lean	0.3	34.3
9-68a	Pork and separable fat/Loin, separable lean	0.3	35.9
8-153c	Tuna/Canned with/Oil, solids and liquid	0.3	37.5
8-54	Skipjack/Katsuo-bushi, dried strip	0.3	39.1
7-32c	Miso/Rice-koji Miso/Dark yellow type	0.2	40.6
8-95a	Pacific saury/Raw/Uncooked	0.2	42.1
16-30c	Other beverages/Coffee/Instant	0.2	43.5
8-206a	Squid and cuttlefish/Raw/Uncooked	0.2	44.9
14-10a	Shimeji/Shirotamogitake, raw/Uncooked	0.2	46.2
17-3a	Seasonings/Shoyu, soy-sauce/Koikuchi	0.2	47.3
9-49b	Chicken and separable fat/Thigh/Broiler	0.2	48.4

^1^Food codes and descriptions correspond to those of the Standard Tables of Food Composition, 4th revised edition in Japan by Science and Technology Agency.

^2^Data on subjects in Ishikawa PHC (14-day data) were counted twice for (28-day data).

**Table 24.** Cumulative % contribution of the top 20 foods for vitamin C assessed by DR

Food code^1^	Description^1^	mg/day	Cumulative^2^ percent
Men (n=102)			
12-24a	Cabbage/Head/Raw	11.9	9.0
12-117a	Spinach/Leaves/Raw	7.4	14.6
13-26a	Japanese Persimmons/Raw fruit/Hard type	6.9	19.9
2-11a	Potatoes/Tuber/Raw	6.2	24.6
12-92a	Bitter gourd/Fruit/Raw	6.2	29.2
16-21b	Green tea/Sencha, common grade/Infusion	5.9	33.7
12-114a	Broccoli/Head/Raw	5.8	38.1
12-56a	Daikon, Japanese radish/Root/Raw	4.9	41.7
13-17b	Satsuma mandarins	4.2	44.9
12-108a	Sweet pepper/Fruit/Raw	4.0	47.9
12-85	Tomatoes/Fruit	3.5	50.6
12-18a	Pumpkin and squash/Squash/Raw	3.4	53.2
16-35	Other beverages/Powdered soft drinks	3.4	55.7
12-98b	Nozawana/Leaves/Salted	3.0	57.9
12-101a	Chinese cabbage/Head/Raw	2.6	59.9
12-32a	Komatsuna/Leaves/Raw	2.1	61.5
13-33	Guavas/Raw fruit	2.0	63.0
12-25a	Cucumber/Fruit/Raw	2.0	64.5
13-6	Strawberries/Raw fruit	1.6	65.7
9-86c	Pork products/Ham/Loin	1.5	66.9
Women (n=113)			
12-24a	Cabbage/Head/Raw	10.5	7.5
13-26a	Japanese Persimmons/Raw fruit/Hard type	8.8	13.8
12-117a	Spinach/Leaves/Raw	7.6	19.2
12-114a	Broccoli/Head/Raw	6.4	23.8
13-17b	Satsuma mandarins	5.9	28.0
16-21b	Green tea/Sencha, common grade/Infusion	5.9	32.3
2-11a	Potatoes/Tuber/Raw	5.7	36.3
13-33	Guavas/Raw fruit	5.1	40.0
12-85	Tomatoes/Fruit	4.7	43.4
12-92a	Bitter gourd/Fruit/Raw	4.5	46.6
12-56a	Daikon, Japanese radish/Root/Raw	4.3	49.7
12-18a	Pumpkin and squash/Squash/Raw	4.3	52.7
12-108a	Sweet pepper/Fruit/Raw	3.6	55.3
13-6	Strawberries/Raw fruit	3.1	57.5
16-35	Other beverages/Powdered soft drinks	3.0	59.7
12-98b	Nozawana/Leaves/Salted	2.5	61.4
12-101a	Chinese cabbage/Head/Raw	2.4	63.1
12-32a	Komatsuna/Leaves/Raw	2.1	64.6
2-5a	Sweet potatoes/Tuber/Raw	2.1	66.1
12-25a	Cucumber/Fruit/Raw	2.0	67.5

^1^Food codes and descriptions correspond to those of the Standard Tables of Food Composition, 4th revised edition in Japan by Science and Technology Agency.

^2^Data on subjects in Ishikawa PHC (14-day data) were counted twice for (28-day data).

**Table 25.** Cumulative % contribution of the top 20 foods for cholesterol assessed by DR

Food code^1^	Description^1^	mg/day	Cumulative^2^ percent
Men (n=102)			
10-5a	Chicken egg/Whole egg/fresh	179.3	42.9
8-206a	Squid and cuttlefish/Raw/Uncooked	34.2	51.1
11-2	Liquid milk/Ordinary liquid milk	11.5	53.9
8-81	Salmon/Salted roe	7.2	55.6
8-218	Spiny lobster, raw	6.8	57.2
8-150a	Tuna/Bluefin tuna, raw/Lean meat	5.8	58.6
9-49b	Chicken and separable fat/Thigh/Broiler	5.0	59.8
9-47b	Chicken and separable fat/Breast	4.9	61.0
8-118a	Salted pollack roe/Uncooked	4.5	62.1
8-78a	Mild salted chum salmon/Uncooked	4.2	63.1
9-70a	Pork/Belly without separable fat	3.9	64.0
10-14	Chicken egg/Tamago-dofu	3.6	64.9
8-95a	Pacific saury/Raw/Uncooked	3.4	65.7
8-84a	Mackerel/Raw/Uncooked	3.4	66.5
9-80	Swine (Sweetbreads)/Liver	3.2	67.3
8-77	Salmon/Chum salmon, raw	3.2	68.1
9-13b	Beef and separable fat/Frank, Short plate	3.1	68.8
4-46	Western-style confectioneries/Shortcake	3.1	69.5
8-109	Sea breams/Crimson sea bream, raw	3.0	70.3
9-68a	Pork and separable fat/Loin, separable lean	2.9	71.0
Women (n=113)			
10-5a	Chicken egg/Whole egg/fresh	149.9	42.1
8-206a	Squid and cuttlefish/Raw/Uncooked	24.3	48.9
11-2	Liquid milk/Ordinary liquid milk	13.2	52.6
8-81	Salmon/Salted roe	6.1	54.3
4-46	Western-style confectioneries/Shortcake	6.1	56.0
8-218	Spiny lobster, raw	5.2	57.5
4-44	Foreign-style confectioneries/Cupcake	4.8	58.8
9-49b	Chicken and separable fat/Thigh/Broiler	4.8	60.2
4-7	Japanese-style confectioneries/Kasutera	4.6	61.5
8-78a	Mild salted chum salmon/Uncooked	3.9	62.5
9-47b	Chicken and separable fat/Breast	3.8	63.6
8-118a	Salted pollack roe/Uncooked	3.7	64.7
9-70a	Pork/Belly without separable fat	3.7	65.7
8-150a	Tuna/Bluefin tuna, raw/Lean meat	3.7	66.7
10-14	Chicken egg/Tamago-dofu	3.3	67.7
8-95a	Pacific saury/Raw/Uncooked	2.9	68.5
8-77	Salmon/Chum salmon, raw	2.8	69.2
9-55	Chicken (Sweetbreads)/Liver	2.6	70.0
8-84a	Mackerl/Raw/Uncooked	2.5	70.7
4-48b	Confectioneries/Cake doughunt	2.3	71.3

^1^Food codes and descriptions correspond to those of the Standard Tables of Food Composition, 4th revised edition in Japan by Science and Technology Agency.

^2^Data on subjects in Ishikawa PHC (14-day data) were counted twice for (28-day data).

## DISCUSSION

A wide variation was observed among the 4 areas both for food and nutrient intake levels at the population level. However, mean intakes of energy and the selected 7 nutrients were not markedly different from the mean values observed in the NNS during a similar period: the difference was within 10% for most of the nutrients compared. The area difference was less marked when energy-density values were used than when the crude values were used in the analysis.

Reports on seasonal differences in nutrient and food group intakes have been few in Japanese populations.^6^ Our data revealed a significant seasonal difference for more than a few nutrients and food groups in both crude and energy-density values. These findings were in general agreement with the previous reports in different areas of Japan.^6^ The data indicated the importance of considering seasonal differences in food group intakes and some specific nutrients such as carotene and vitamin C, where long-term dietary habits are concerned. DRs from the Okinawa (Ishikawa PHC) area were collected only in winter and summer becaouse the seasonal variation was expected to be minimal in a sub-tropical climate. Thus, seasonal differences in dietary habits in the Ishikawa PHC area were not investigated in the present study. When the intakes were compared between winter and summer including the data of the Ishikawa PHC area, the results were virtually consistent.

The top 20 foods contributing to intake of each nutrient in DRs were included in the FFQ food items, except for some which contributed to alcohol, carbohydrate, and sodium intakes. The lowest cumulative percentage among the top 20 foods was observed in potassium in men (46%) and in women (44%). The cumulative percentage among the top 20 foods was slightly lower in women than in men in most of the nutrients examined. The results were generally similar to those previously reported for a different population in Japan.^7^^,^^8^
